# Four new species of coral gobies (Teleostei: Gobiidae: *Gobiodon*), with comments on their relationships within the genus

**DOI:** 10.11646/zootaxa.3709.4.1

**Published:** 2013-09-06

**Authors:** Juergen Herler, Sergey V. Bogorodsky, Toshiyuki Suzuki

**Affiliations:** 1 Department of Integrative Zoology, Faculty of Life Sciences, University of Vienna, Althanstrasse 14, A-1090 Vienna, Austria. Juergen.Herler@univie.ac.at; 2 Station of Naturalists, S. Tyulenina ul. 13–29, Omsk, 644090, Russia. ic187196@yandex.ru; 3 Kawanishi-midoridai Senior High School, 1–8 Kouyoudai, Kawanishi, Hyogo 666-0115, Japan. trimma-toshiyuki@hop.ocn.ne.jp

**Keywords:** taxonomy, phylogeny, Red Sea, 12S rRNA, 16S rRNA, coral reefs

## Abstract

Four new species of the coral-associated gobiid genus *Gobiodon* were discovered in the Red Sea. Although several of these species are common not only in the Red Sea but also in the Indian and western Pacific Ocean, they have not been described before. Detailed descriptions of the four species are based on morphological and molecular genetic (mitochondrial 12s and 16s rRNA) investigations. The new species, like most species of the genus, lack scales and have species-specific life colouration. *Gobiodon bilineatus* sp. nov. is the closest relative to *G. quinquestrigatus* (Valenciennes) and of *G*. sp. D (Munday *et al*.), and has five distinct, blue lines on the head as juveniles and subadults, which disappear in adults, and which are often uniformly orange-red with two distinct, vertical blue lines through each eye. *Gobiodon irregularis* sp. nov. has been confused with the former new species in the past, and is closely related to *G. oculolineatus* Wu, but is unmistakable in live colouration. Juveniles are characterised by a transparent body, red bars on the head with bluish to greyish interspaces, and irregular red lines and dots on the nape and dorsally on the body. Adults are usually uniformly brown or green-brown, with only remnants of the bars through the eye and below the orbit. *Gobiodon ater* sp. nov. is a small, entirely black species and can be easily confused with other black species, although it is genetically clearly distinct from *G. ceramensis* Bleeker and its black relatives. *Gobiodon fuscoruber* sp. nov. is likely to be the closest relative of *G. ater* sp. nov., but is uniformly reddish-brown or brown, has bright median fin margins (at least in the Red Sea), and grows considerably larger than *G. ater*. It has been genetically determined that *G. fuscoruber* sp. nov. is identical with an Indian Ocean/western Pacific species that has been called *G. unicolor* Castelnau by several authors. However, examination of the holotype of *G. unicolor*, including the original description, revealed that the type species and original description are clearly different from the species frequently called *G. unicolor*. The holotype resembles *G. histrio* (Valenciennes) and the name *G. unicolor* must therefore be considered a junior synonym of *G. histrio*. As a consequence, a new name for this species is provided.

## Introduction

The coral-associated Indo-Pacific gobiid genus *Gobiodon* Bleeker 1856 comprises more than 20 valid species (Eschmeyer 2012). Although this genus of small, cryptic coral gobies is quite well-known among reef fishes, new species have been described recently (Winterbottom & Harold 2005; Suzuki *et al*. 2012), and many more are known but remain undescribed (Munday *et al.* 1999; Akihito *et al.* 2002; Senou *et al.* 2004; Herler & Hilgers 2005). The taxonomy of *Gobiodon* is difficult for several reasons. On the one hand, taxonomists are challenged by the high diversity of largely similar species, the large distribution areas of some species and corresponding geographic variation, as well as the lack of suitable diagnostic characters in external morphology. On the other hand, a series of unsatisfactory species descriptions, especially those from the 19^th^ century, make reliable identification and species designation difficult. The problem is increased by preservation, which rapidly fades life colour, a key characteristic for species identification. The taxonomy of this genus is therefore far from being resolved and several species will require redescription. Therefore, new species descriptions must be carried out cautiously to avoid further confusion in the taxonomy of *Gobiodon*.

Herler and Hilgers (2005) discovered three species in the Red Sea that were not known from this area before. As they could not be assigned to any previously described species, they were provisionally designated as *Gobiodon* sp. 1, sp. 2 and sp. 3. Recent molecular genetic examinations revealed that one of these species (sp. 1) are two different species, and therefore four new species are here described. In order to avoid further confusion, examination of and comparison with different type and non-type material and extensive genetic investigations of taxa from various regions were carried out. The present study also provides information on the life colouration, habitats and distribution, and molecular genetic evidence for species delimitation and novelty. Morphological descriptions are mainly based on simple features such as species-specific life colouration, fin meristics and traditional and geometric morphometrics; these results are combined with molecular genetic data in order to enable also non-taxonomists, ecologists and geneticists to more easily identify the species, even in the field.

## Materials and methods

Life colouration was recorded from specimens that were anaesthetised with a dash of clove oil (diluted in ethanol) added to the sea water. Preserved colouration was recorded after preservation in 70% ethanol, following fixation in 5% formalin. Narcotised and preserved specimens were scanned with a flatbed scanner (Epson V300) following the procedures of Herler *et al.* (2007). In preserved specimens, the skin mucus layer was removed prior to scanning, and the preserved colouration of several specimens therefore appears clearer than in Herler and Hilgers (2005). Sex determination in the bi-directionally sex-changing genus *Gobiodon* is only safely possible in pairs during breeding, but difficult when fishes are collected as single adults or out of breeding season. The sex for several specimens is thus not given. For a more detailed description of the colouration, selected identified specimens were included as additional material and are shown alive, as freshly collected or preserved. Further additional material is represented by specimens that were DNA-sequenced.

### Morphometrics

Traditional morphometrics: body measurements follow the methods of Winterbottom and Harold (2005), as well as caudal fin length (from end of hypural plates to tip of longest medial caudal ray). Fish size is given as standard length (SL) or total length (TL). Body proportions were measured under a binocular microscope with an electronic calliper to the nearest 0.01 mm, after the skin mucus layer was removed. Thus, some measurements may differ from the values presented by Herler and Hilgers (2005), where the mucus was not removed. Morphometric measurements are provided for holo- and paratypes. A principal components analysis of body proportions (all expressed as % SL) of type specimens larger than 20 mm SL was carried out with the program PAST (Hammer *et al*. 2001), version 2.12, to explore species-specific phenotypic characters of the four new species. Loadings of variables onto principal components were investigated to identify the most important discriminating body proportions.

### Geometric morphometrics

In addition to traditional morphometrics, we performed a total body shape analysis employing a landmark system and a multivariate statistical analysis of landmark coordinates. This analysis should prove that species can be discriminated morphometrically, and it should in particular aid in difficult species assignments, such as for the doubtful holotype of *G. unicolor*. Therefore, digital lateral images of 111 ethanolpreserved specimens (33 *G. histrio*, 9 *G. bilineatus* sp. nov., 17 *G. irregularis* sp. nov., 16 *G. ater* sp. nov., 25 *G. fuscoruber* sp. nov. from the Red Sea, 10 *G. fuscoruber* sp. nov. from the western Pacific and the holotype of *G. unicolor*) were used. *Gobiodon histrio* was included for comparison because we assume that the holotype of *G. unicolor* is identical with *G. histrio* (Valenciennes 1837) based on several other features (described in detail below). From each specimen coordinates of the following 15 landmarks were taken: 1: orbital center, 2: snout tip, 3: D1 origin, 4: D2 origin, 5: end of D2, 6: dorsal origin of C, 7: median point of C origin, 8: ventral origin of C, 9: end of A, 10: origin of A, 11: origin of V, 12: ventral origin of P, 13: dorsal origin of P, 14: dorsal insertion of gill cover, 15: posterior end of upper lip. In addition, 6 outline semi-landmarks (3 along the forehead, between landmarks 2 and 3, and 3 along the breast, between landmarks 2 and 11, all in equidistant position) were employed. Landmarks were set using the programs tpsUtil (Rohlf 2010a) and tpsDig2 (Rohlf 2010b). Landmark data were analysed via principal components analysis (PCA) of the partial warps of Procrustes coordinates using the program tpsRelw (Rohlf 2010c), including the application of a sliders-file for the 6 semi-landmarks. Plots of PCA-scores and MANOVA on the first five PCs (comprising more than 5% of the total variance) of the six species with n>1 were carried out in the program PAST (Hammer *et al*. 2001), version 2.12. Finally, a discriminant function analysis was performed on the first 6 PCs for all 111 specimens in SPSS 19 for Windows.

### Meristics

Fin-ray counts are provided for type specimens. Spines (solid elements) are indicated with Roman numbers, soft rays (bilaterally paired, segmented elements) in Arabic numbers. The most frequent value is written in bold, whereas values of holotypes are underlined in each description. Abbreviations: A—anal fin, C—caudal fin, D1 and D2—first and second dorsal fin, P—pectoral fin, V—pelvic fin (disc). Vertebrae were counted in radiographed type specimens: *G. bilineatus* sp. nov.: 1 spec., 35.7 mm SL (holotype NMW 95077); *G. fuscoruber* sp. nov.: 3 spec., 29.2–37.6 mm SL (holotype NMW 95079 and paratypes BMNH 1951.1.16.555 and 556). Gill rakers were counted on the first right gill arch from several cleared and stained non-types.

### Molecular genetics

Combined 12S and 16S mitochondrial rDNA sequences were used for the molecular genetic analysis. Sequences for most taxa were taken from GenBank deposits of Harold *et al*. (2008) and Herler *et al*. (2009). In addition, new specimens from different species and regions were sequenced (see Herler *et al*. (2009) for sequencing procedures), with a focus on the four new species (see below for details), and including *G.* cf. *fulvus* Herre 1927 (we are cautious with applying the name *G. fulvus* here, because *G. fulvus* of Winterbottom and Emery (1986) seems to be different from Herre’s description), which also has not been sequenced before. Since replicate specimens of the four new species were genetically almost identical within regions, only one to two representatives of each species from each region were included in a neighbor-joining analysis of all species of *Gobiodon* for which sequences were available. These include, apart from the four new species, 18 taxa from the western Pacific, the Indian Ocean and/or the Red Sea. Eleven taxa (*G. ater* sp. nov., *G. axillaris* De Vis 1884, *G. bilineatus* sp. nov., *G.* cf. *bilineatus, G. citrinus* (Rüppell 1838), *G.* cf. *fulvus, G. fuscoruber* sp. nov., *G. histrio* (Valenciennes 1837), *G. quinquestrigatus* (Valenciennes 1837), *G. rivulatus* (Rüppell 1830) and *G*. sp. D sensu Munday *et al*. (1999)) were sampled from more than one region to also reveal geographic variation (Table 1; Fig. 11). The genus *Paragobiodon* (*P. echinocephalus* and *P. xanthosoma*) was included as the sister genus to *Gobiodon*. GenBank accession numbers for all specimens and both markers are provided in Table 1. Alignment, calculation of genetic distances (*p*-distances) and phylogenetic analyses were performed with MEGA 5.05 (Tamura *et al*. 2011) for PC. According to a model test, a Kimura-2P-model was used for a NJ-analysis with 1000 bootstrap replicates. The combined sequences had a length of 853 base pairs after alignment, which were analysed.

### Collection abbreviations

BMNH—British Museum of Natural History, London.

CH—Collection Herler.

MNHN—National Museum of Natural History, Paris.

NMW—Natural History Museum, Vienna.

OMNH— Museum of Natural History, Osaka.

PMR—Natural History Museum, Rijeka.

SMF—Senckenberg Museum, Frankfurt.

## Taxonomy

### *Gobiodon bilineatus* sp. nov

Two-lined Coralgoby

**Figure 1:**
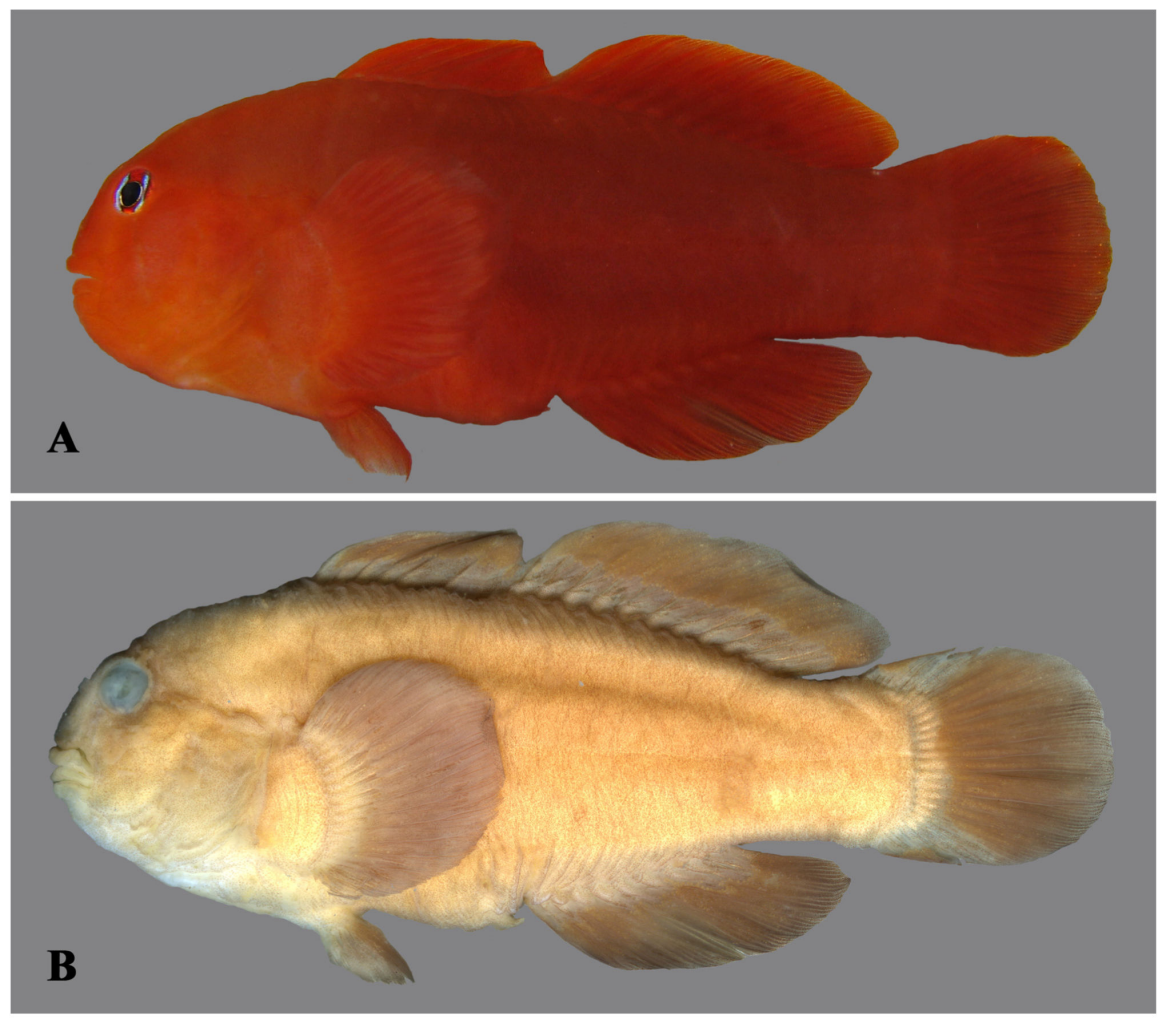
*Gobiodon bilineatus* sp. nov., adult, holotype NMW 95077, 35.7 mm SL, Dahab, Egypt, Red Sea. **A.** freshly collected; **B.** ethanol-preserved. Photos by J. Herler.

**Figure 2:**
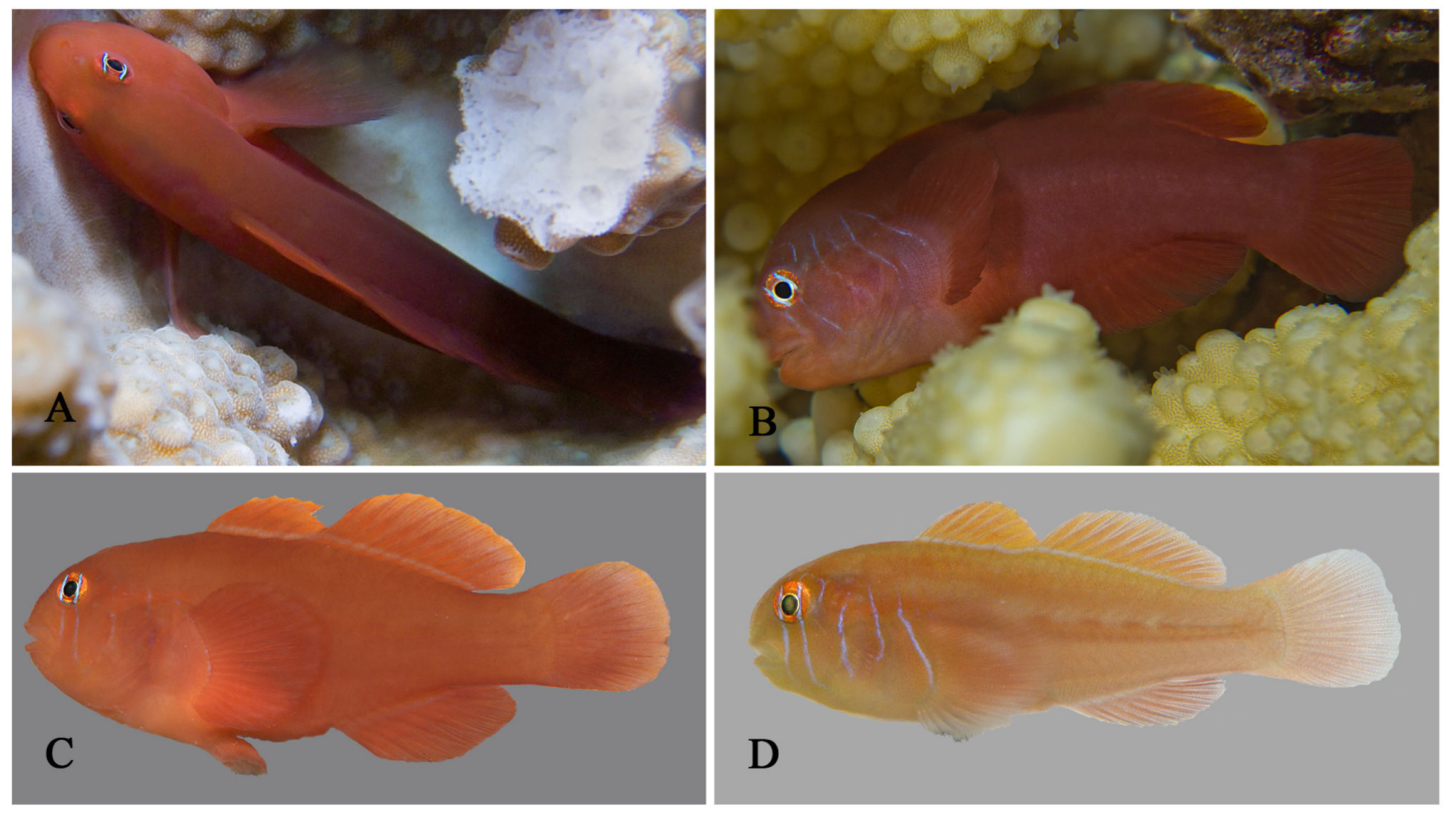
*Gobiodon bilineatus* sp. nov. **A.** alive adult, PMR VP2234, 32.7 mm SL, Hanish Island, Yemen, Red Sea; **B.** alive subadult, PMR VP3200, 23.7 mm SL, Sharm el Moya, Egypt, Red Sea; **C.** freshly collected adult, paratype BMNH 2006.10.6.1, 29.4 mm SL, Dahab, Egypt, Red Sea; **D.** freshly collected juvenile, CH 232-41-063, 18.0 mm SL, Dahab, Egypt, Red Sea. Photos by S. V. Bogorodsky (A, B), J. Herler (C, D).

Figs. 1, 2, 10 and 11; Tables 1, 2, 3 and 10

*Gobiodon* sp. 1 Herler and Hilgers 2005 (part): 120, Figs. 13c–e; Bogorodsky *et al*. 2010: 122, Figs. 3, 4.

#### Holotype

NMW 95077 (CH 232–41–011 in Herler & Hilgers, 2005), male 35.7 mm SL, Gulf of Aqaba, Egypt, Dahab, “Islands” (28°28′38.5″ N, 34°30′47.1″ E), coll. M. Dirnwoeber, 17 April 2004.

#### Paratypes

Four specimens. NMW 95563, 32.9 mm SL, Gulf of Aqaba, Egypt, Dahab, “Soliman Reef” (28°28′47.0″ N, 34°30′51.8″ E), coll. J. Herler, 2 May 2010. NMW 95564, juvenile, 22.7 mm, same data as NMW 95563. MNHN 2012-0262, 34.9 mm SL, other data same as NMW 95563. BMNH 2006.10.6.1, 29.4 mm SL, 30 May 2010, other data same as NMW 95563.

#### Additional material

CH 232-41-063 (DNA sample + photograph), juvenile, 18.0 mm SL, Egypt, Dahab, coll. J. Herler, 30 May 2012; PMR VP2234, 32.7 mm SL, Yemen, Hanish Island, coll. S.V. Bogorodsky, 23 October 2009; PMR VP3200, 23.7 mm SL, Egypt, Sharm el Sheikh, Sharm el Moya, coll. S.V. Bogorodsky, 04 July 2011.

#### Comparative material

*Gobiodon* cf. *bilineatus*: uncatalogued (DNA sample; Fig. 9A), 28.9 mm SL, Maldives, Kagi Island, coll. J. Herler, 16 March 2007; uncatalogued (DNA sample; Fig. 9B), 22.9 mm SL, southern Taiwan, Kenting, coll. J. Herler, 06 December 2008. *Gobiodon prolixus*: holotype, ROM 73338 (Fig. 9C), male, 26.2 mm, Vietnam, Nha Trang, coll. R. Winterbottom, W. Holleman, B. Hubley, M. Burridge, M. Winterbottom and N. Vij, 27 May 2002; ROM 84987, female, 19.6 mm, Yemen, Hanish Island, coll. S.V. Bogorodsky, 23 October 2009; PMR VP2233, female, 18.9 mm SL, Yemen, Hanish Island, coll. S.V. Bogorodsky, 23 October 2009. *Gobiodon* sp. D sensu Munday *et al*., (1999): uncatalogued (DNA sample; Fig. 9D), 29.9 mm SL, Maldives, Hembadhu Island, coll. J. Herler, 18 March 2007. *Gobiodon quinquestrigatus*: uncatalogued (DNA sample, Fig. 9E), 28.7 mm SL, southern Taiwan, Kenting, coll. J. Herler, 04 December 2008.

#### Diagnosis

Dorsal-fin rays VI + I,10–11; anal-fin rays I,8–9 (usually 9); head and body naked; body slightly compressed, relatively elongate (body depth at pelvic-fin origin 36–40% SL), dorso-ventrally symmetrical; distance between D1 insertion and dorsal insertion of pectoral-fin 53–71% of head length; head rounded, with upper lip curved; mouth small, upper jaw extending to anterior margin of the eye; no groove between isthmus and interopercle; caudal peduncle deep (minimal depth 16.3–17.9% SL); caudal fin relatively short (21.7–23.2% SL). Juveniles and subadults light greenish or reddish with five vertical blue lines on head; adults usually uniformly orange-red or dark red, sometimes with remnants of lines on the head but always with two distinct bluish lines through the eyes, sometimes extending ventrally to suborbital area; dorsal fins and anal fin often with a narrow light bluish band along bases, fading with growth.

#### Description

(based on 5 types and several non-type specimens (for osteology)). Head and body only slightly compressed. Body dorso-ventrally symmetrical, head rounded, caudal peduncle deep. Body proportions and meristics for types are provided in Tables 2 and 3, respectively. Dorsal-fin rays VI + I,**10**–11 (10:3, 11:2); anal-fin rays I,8–**9** (8:1, 9:4); pectoral-fin rays **19**–20 (19:3, 20:2); pelvic-fin rays I,5 (all specimens); caudal fin with 15–17 segmented and branched rays; disc short (not reaching anus) and cup-shaped, with significant frenum between spines. First dorsal fin rounded and as high as second dorsal in juveniles, but shorter than D2 in adults. Vertebral column with 10 precaudal and 16 caudal vertebrae, including urostyle. No scales. Gill opening less wide than pectoral-fin base, ending ventrally in opposite of 3^rd^ or 4^th^ lower pectoral-fin ray. Gill rakers 1–2 + 7–8. No obvious groove between interopercle and isthmus. Mouth terminal, slightly oblique, bending downwards. Upper jaw reaching to below anterior orbital margin. Upper lip usually curved, slightly extends in front of snout. One outer row of 5 to 12 larger, slightly recurved teeth in upper and lower jaw, and increasing in size towards symphysis. Several rows of small, slender and recurved teeth in both jaws behind the outer row. Lower jaw with a pair of large, postsymphysial canines on each side, one often smaller or absent, probably due to tooth loss and replacement. Anterior and posterior nasal openings at the end of short tubes. Head sensory canals typical for *Gobiodon* (Winterbottom & Harold 2005), with anterior oculoscapular (pores NA (paired), AI, PI (unpaired), SO, AO and IT (paired)) and preopercular (three pores on each side) canals present.

#### Life colouration

Juveniles reddish or greenish with five vertical blue lines across head (Fig. 2D), first and second through eye and cheek, third and fourth across anterior and posterior margin of opercle, and fifth across pectoral-fin base. Adults mostly uniformly bright orange-red (Figs. 1A, 2A). Smaller adults sometimes with remnants of blue lines on the head (Figs. 2B, C). Large adults mostly only with two distinct bright blue lines through eye. A narrow, pale bluish band sometimes visible along the dorsal-fin bases.

#### Preserved colouration

Uniformly light or dark brown. Lines on head and/or through eyes diminished (Fig. 1B).

#### Molecular genetics

The present analysis includes two newly sequenced specimens from the Red Sea because Herler *et al*. (2009) sequenced only specimens that have now been assigned to *G. irregularis* sp. nov. In addition, similar-looking specimens from the Maldives and Taiwan were included. See Table 1 for Genbank accession numbers. The genetically closest described species to *G. bilineatus* sp. nov. is *G. quinquestrigatus* (Valenciennes 1837) (Fig. 11). The *p*-distance between the two species is 0.033 (genetic distance >3%).

#### Habitat

*Gobiodon bilineatus* sp. nov. most frequently occupies the reef slope and fore reef areas. It is often found in large colonies of *Acropora samoensis* but is also present in *A. secale* and *A. gemmifera* in the northern Red Sea.

#### Distribution

This species was found in the Gulf of Aqaba, in the northern Red Sea main basin (near Marsa Alam, Egypt and Al Wajh, Saudi Arabia) and in the southern Red Sea (Dahlak Archipelago, Eritrea; Hanish Island, Yemen). Its distribution range may extend to the Indian and western Pacific Ocean (see Remarks and Fig. 9).

#### Etymology

This species is named for its two distinct, bright blue lines through the eye, which is the only distinct colour pattern that remains in the largest adults when alive. The name “bilineatus” is derived from the latin words “bi”, meaning two, and “linea” for line. Suggested common name: Two-lined Coralgoby. Allen and Erdmann (2012) already used the common name Twoline Coralgoby for their *G.* sp. 2, but they actually show *G. fulvus* sensu Winterbottom and Emery (1986), a species known under the common name Brown Coralgoby.

#### Remarks

Herler and Hilgers (2005) mistook two genetically but also morphologically distinct species for one: *G. bilineatus* and *G. irregularis* sp. nov. were erroneously pooled as *Gobiodon* sp. 1. The authors considered *G. irregularis* sp. nov. as the juvenile/subadult form of *G. bilineatus*. Only our recent genetic investigations revealed that the juveniles of *G. bilineatus* are actually rather uniformly coloured with 5 blue lines on the head as the only distinctive colour pattern. Therefore, very small (< 1 cm SL) *G. bilineatus* may be almost indistinguishable from similar-sized *G. rivulatus, G. quinquestrigatus, G*. sp. D sensu Munday *et al*. (1999) or *G. prolixus* Winterbottom and Harold (2005), with at least the three former also being genetically close taxa (Fig. 11). There are also no unique fin meristic features which could help to discriminate between them (see also Herler & Hilgers 2005, and Winterbottom & Harold 2005). Although the lesser body depth and shorter D1-P1-distance of *G. prolixus* (28.2–35.5% SL and 41.3–50.4% of head length, respectively, vs 36–40% SL and 53.4–70.4% of head length in *G. bilineatus*) may help distinguish this species when adult (although differences in colouration will then also be evident), it is doubtful that these body proportions are also distinct in juveniles. Adult *G. bilineatus*, however, are distinct in that the 5 vertical blue lines disappear with growth, and in their bright orange-red colour. As mentioned, the three most similar and also genetically closest species are *G. quinquestrigatus, G*. sp. D sensu Munday *et al*. (1999) and *G. rivulatus*. According to Duchene *et al.* (2013), the two former species are genetically distinct. All four species can be distinguished by slight differences in life colour, although field identification may be very difficult, in particular between the two former species and *G. bilineatus*. *Gobiodon rivulatus* is most easily distinguished by the three or more additional, shorter lines on the head, which are positioned in the interspaces of the typical five long lines on the head, and by the many more irregular lines on the body (particularly visible in lighter coloured forms). By contrast, the other three species have only five bluish lines on head (sometimes a very short sixth line behind the upper pectoral-fin base), all in the same position (Figs. 2B, C, 9D, E). When lines on the head disappear in adults of *G. bilineatus*, this species is characterised by a uniformly orange-red colour, including the fins (Figs. 1A, 2A). *Gobiodon quinquestrigatus* differs by its very distinct and bluish lines on head, which remain in adults on the otherwise orange-brown head, and by its dark brown body colouration. *Gobiodon* sp. D sensu Munday *et al*. (1999) has a reddish body colouration, less distinct bluish lines on head, and brown fins that are darker than the body. The latter species and juveniles and subadults of *G. bilineatus* are similar to each other in that a narrow bright bluish band is frequently present along the dorsal-fin bases. This feature is less distinct or absent in *Gobiodon quinquestrigatus*. It is unclear whether the Indian Ocean (Maldives) and western Pacific (Taiwan) populations (designated as *G.* cf. *bilineatus* herein) should be considered as *G. bilineatus* or as a very closely related, but distinct species (Figs. 9, 11). They differ from typical *G. bilineatus* in that the maximum size seems to be smaller (many specimens were observed in the Maldives and collected by JH) and their life colour is much darker (Figs. 9A, B). Note that life colour is usually distinct in different *Gobiodon* species. Although they show genetic distances (Table 10) of < 1.4%, we refrained from including this material in the species description because the distinct life colouration strongly indicates that it is a separate species.

### *Gobiodon irregularis*, sp. nov.

Rufous Coralgoby

**Figure 3:**
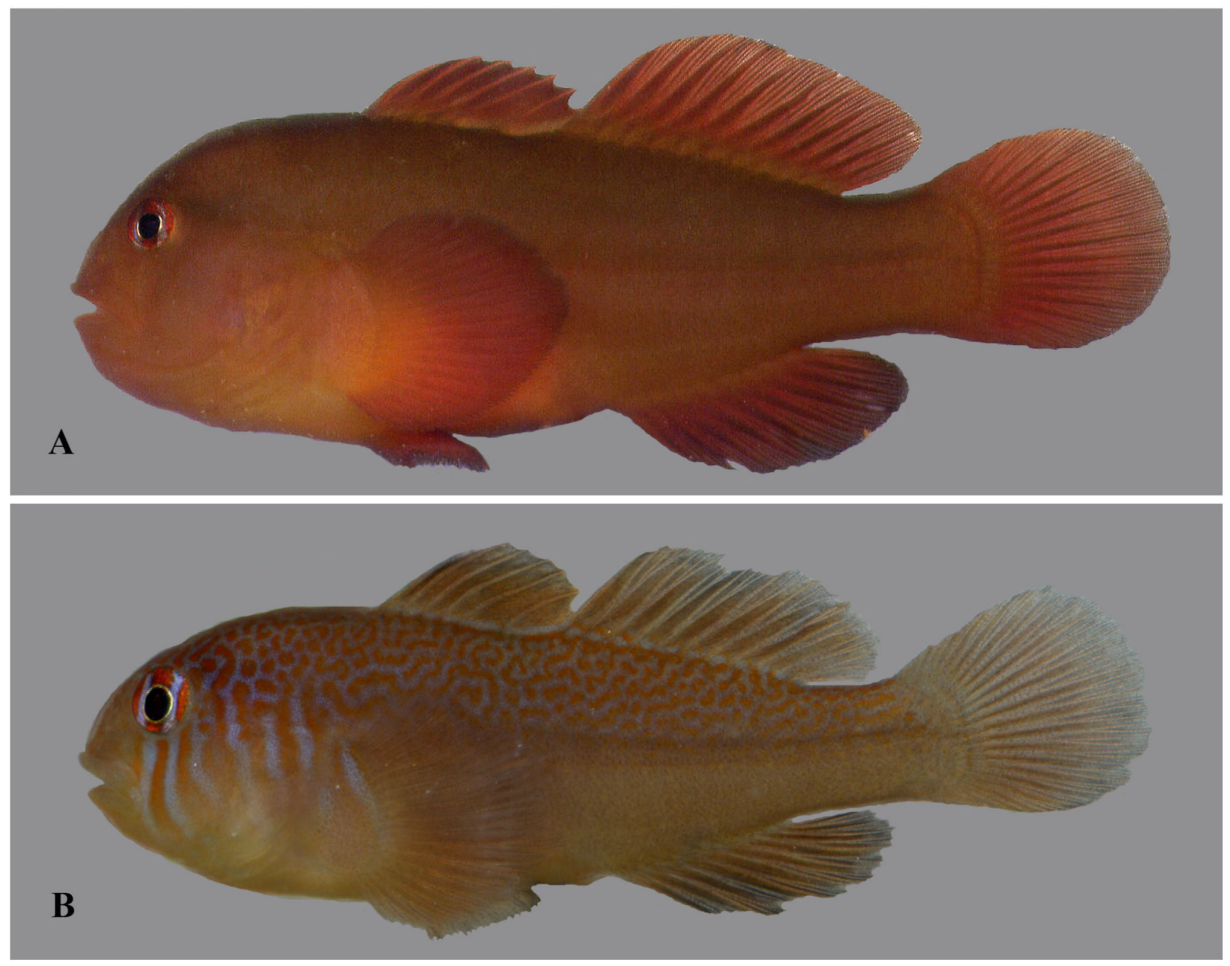
*Gobiodon irregularis* sp. nov. **A.** freshly collected adult, holotype, NMW 95078, 30.1 mm SL, Dahab, Egypt, Red Sea. **B.** freshly collected subadult, CH 232-41b-057, 22.3 mm SL, Dahab, Egypt, Red Sea. Photos by J. Herler.

**Figure 4:**
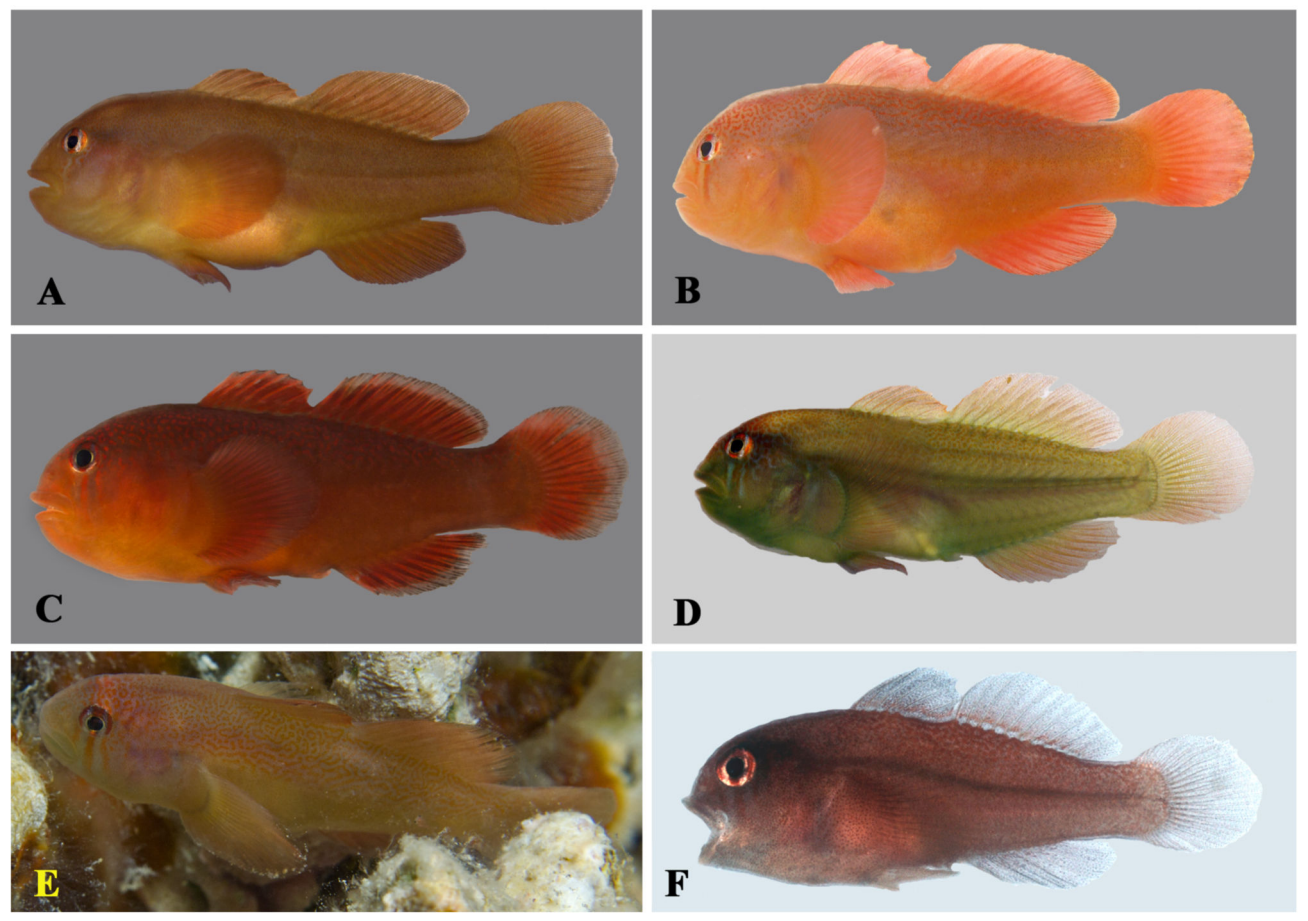
*Gobiodon irregularis* sp. nov. **A.** freshly collected adult, paratype MNHN 2006-1699, 32.3 mm SL, Dahab, Egypt, Red Sea; **B.** freshly collected adult, CH 232-41-020, 31.2 mm SL, Dahab, Egypt, Red Sea; **C.** freshly collected adult, SMF uncatalogued, 27.6 mm SL, Farasan Archipelago, Red Sea; **D.** freshly collected subadult, paratype NMW 95565, 23.4 mm SL, Dahab, Egypt, Red Sea; **E.** alive subadult, PMR VP3201, 23.5 mm SL, Sharm el Moya, Egypt, Red Sea; **F.** freshly collected juvenile, SAIAB 70430, 15 mm SL, Rodrigues, western Indian Ocean. Photos by J. Herler (A, B, D), S.V. Bogorodsky (C, E), P. Heemstra, © SAIAB (F).

Figs. 3, 4, 10 and 11; Tables 1, 4, 5 and 10

*Gobiodon* sp. 1 Herler and Hilgers 2005 (part): 120, Figs. 13a, b; Herler *et al*. (2009): 733, Fig. 4.

#### Holotype

NMW 95078, 30.1 mm SL, Gulf of Aqaba, Egypt, Dahab, “Islands” (28°28′38.50″ N, 34°30′47.10″ E), 11 m, coll. J. Herler, 13 November 2005.

#### Paratypes

Four specimens: NMW 95565, 23.4 mm SL, Gulf of Aqaba, Egypt, Dahab, “Napoleon Reef” (28°28′14.4″ N, 34°30′31.4″ E), 1 m, coll. J. Herler, 18 November 2005. NMW 95566 (CH 232-41-019 in Herler & Hilgers, 2005), 16.1 mm SL, 3 m, 4 May 2004, other data same as holotype. MNHN 2006–1699, 32.3 mm SL, 5 m, 19 November 2005, other data same as holotype. BMNH 2006.10.6.1, 29.6 mm, 3 m, 17 November 2005, other data same as holotype.

#### Additional material

*Gobiodon irregularis*: CH 232-41b-057, 22.3 mm SL, Egypt, Dahab, coll J. Herler, 13 May 2012; CH 232-41-020, 31.2 mm SL, Egypt, Dahab, coll J. Herler, 04 May 2004; SMF, uncatalogued, 27.6 mm SL, Saudi Arabia, Farasan Archipelago, coll. S.V. Bogorodsky, 27 February 2012; PMR VP3201, 23.5 mm SL, Egypt, Sharm el Sheikh, Sharm el Moya, coll. S.V. Bogorodsky, 30 June 2011. *Gobiodon* RW sp. 1 (identified as *G. irregularis*): SAIAB 70430, juvenile, 15 mm SL, Indian Ocean, Rodrigues, coll. P. Heemstra, 22 October 2001.

#### Comparative material

*Gobiodon* cf. *fulvus* (sensu Winterbottom & Emery 1986): uncatalogued (DNA sample), 23.9 mm SL, Maldives, Makunudu, coll. J. Herler, 17 March 2007; uncatalogued (DNA sample), 23.7 mm SL, southern Taiwan, Kenting, coll. J. Herler, 06 December 2008; OMNH P40259 (Fig. 9F), 20.8 mm SL, Japan, Ryukyu Islands, Iriomote Island, coll. T. Suzuki and M. Suzuki, 22 August 2004. *Gobiodon oculolineatus* Wu 1972: OMNH P40260 (Fig. 9G), 22.6 mm SL, Japan, Ryukyu Islands, Okinawa Island, coll. T. Suzuki and M. Suzuki, 04 May 1996.

#### Diagnosis

Dorsal-fin rays VI + I,10-11 (usually 11); anal-fin rays I,9-10 (usually 9); head and body naked; body relatively elongate (depth 35.1–39% SL) and compressed; head slightly pointed in juveniles, becoming more rounded in adults; snout sometimes with a slight hump above upper lip; no groove between isthmus and interopercle; caudal peduncle slender (depth 14.5–15.8% SL); caudal fin relatively long (22.6–24.6% SL). Juveniles and subadults greenish or brownish green with up to seven red bars with bluish interspaces on head and pectoral-fin base, three anteriormost bars also run across eye; nape and dorsal part of body with irregular red lines and small spots; lines and dots usually vanish in adults of more than 3 cm TL; body becomes uniformly brown or reddish brown, usually with remnants of the orbital and suborbital bars.

#### Description

(based on 5 types and several non-type specimens (for osteology)). Head and body compressed. Head relatively deep, posterior part of body slender, caudal peduncle low. Body proportions and meristics for types are provided in Tables 4 and 5, respectively. Dorsal-fin rays VI + I,10–**11** (10:1, 11:4); anal-fin rays I,**9**–10 (9:4, 10:1); pectoral-fin rays 20 (n = 5); pelvic-fin rays I,5 (all specimens); caudal fin with 15-17 segmented and branched rays; disc short (not reaching anus) and cup-shaped with significant frenum between spines. First dorsal fin as high as D2 in juveniles and somewhat triangular, but lower than D2 in adults. No scales. Gill opening less wide than pectoral-fin base, ending ventrally in opposite of 3^rd^ or 4^th^ lower pectoral-fin ray. Gill rakers 0-3 + 7-8. No groove between interopercle and isthmus. Mouth terminal, relatively straight. Upper jaw reaching to about below anterior margin of orbit or to mid-orbit. Upper lip slightly curved. One outer row of up to 10 larger, slightly recurved teeth in upper and lower jaw, and increasing in size towards symphysis. Several rows of small, slender and recurved teeth in both jaws behind the outer row. Lower jaw with a pair of large, postsymphysial canines on each side, one often much smaller or absent, probably due to tooth loss and replacement. Anterior and posterior nasal openings at the end of short tubes. Head sensory canals typical for *Gobiodon* (Winterbottom & Harold 2005), with anterior oculoscapular (pores NA (paired), AI, PI (unpaired), SO, AO and IT (paired)) and preopercular (three pores on each side) canals present.

#### Life colouration

Juveniles greenish with seven broad red bars on head and pectoral-fin base (Fig. 3B): first from orbit to upper lip, second and third through orbit and across cheek, fourth to sixth, wider and somewhat wavy, across opercle, seventh wide across pectoral-fin base; interspaces bluish or greyish; red bars become irregular and wavy on postorbital area; nape and dorsal half of body with red vermiculations; both dorsal fins sometimes with narrow bright bluish band along the base; banded pattern of internal pigment along vertebral column; median fins greenish. Subadults brownish green or brown with small red spots and irregular short lines on nape and dorsal part of body; only the three more distinct red suborbital head bars remain visible (Fig. 4B-E). Adults uniformly reddish brown or brown, including fins (Figs. 3A, 4A), nape and upper half of body covered with dark-brown dots; remnants of orbital bars, extending onto suborbital area, may be visible.

#### Preserved colouration

Specimens uniformly light or dark brown. Bars and dots in juveniles and subadults may be retained. Bars through eyes not visible.

#### Molecular genetics

The present analysis includes the sequences of the specimen with the original number *G*. sp. 1_GA1 (Herler *et al*. 2009) from the Gulf of Aqaba, Red Sea as well as of one of the paratypes (NMW 95565). See Table 1 for Genbank accession numbers. The genetically closest species to *G. irregularis* sp. nov. is *G. oculolineatus* Wu, 1972 (Duchene *et al*. 2013). The *p*-distance based on 12S and 16S rRNA comparisons between these two species is 0.013 (1.3% genetic difference; Herler, unpublished data).

#### Habitat

*Gobiodon irregularis* displays a generalised habitat selection and occupies a great range of *Acropora* corals. It occurs in deeper water regions (lower reef slope and fore reef areas) and is most common in corals such as *Acropora samoensis*, *A. valida* and *A. secale* but was also observed in *A. eurystoma* and *A. pharaonis* in the northern Red Sea.

#### Distribution

This species was found in the Gulf of Aqaba, northern Red Sea main basin (near Marsa Alam, Egypt and Al Wajh, Saudi Arabia) and in the southern Red Sea (Dahlak Archipelago, Eritrea; Farasan Archipelago, Saudi Arabia). It is also known from Rodrigues in the western Indian Ocean (identified as *Gobiodon* RW sp. 1 by Richard Winterbottom from a photo by Phil Heemstra; Fig. 4F).

#### Etymology

This species is named “irregularis” for its variable colouration, in particular its irregular red wavy lines on the head and upper body in juveniles and subadults. Suggested common name: Rufous Coralgoby (colored red-brown when adult).

#### Remarks

The confusion of this species with *G. bilineatus* and grouping with *Gobiodon* sp. 1 (Herler & Hilgers 2005) was mainly caused by the very similar adult colouration, although the body shape (in particular the rounder head and deeper caudal peduncle in *G. bilineatus*) and lines on the head are different in adult fishes. Also, D2, A and P fin-ray counts are on average higher in *G. irregularis*, but the overlapping range of values does not permit definite identification. As confirmed by mitochondrial DNA investigations, the two species are genetically very distinct; the same holds true for the juvenile and subadult colouration of both. Although body shape differences are recognizable, geometric morphometric analysis failed to reveal statistically significant shape differences between the two species (MANOVA on the first 5 principal components: p = 0.08), but this may merely reflect the very low sample number, especially of *G. bilineatus*. Discrimination between *G. irregularis* and *G. bilineatus* can possibly be based on life colouration, at least among Red Sea specimens. Juveniles and subadults may be distinguished by the five thin blue lines on the head and the uniformly coloured body in *G. bilineatus*, versus the broader irregular red bars with bluish/greyish interspaces below the eye and the irregular lines and dots on the body of *G. irregularis*. In adults, lines on the head or bars may vanish in both species, but there are always two bright bluish lines running across the eye in *G. bilineatus*, which are far less distinct in *G. irregularis*. Moreover, body colouration is bright orange to orange-red in *G. bilineatus* and rather brownish to red-brown in *G. irregularis*, but this varies: the Indian Ocean/western Pacific populations, which are genetically very close to Red Sea *G. bilineatus* (Fig. 11), have a darker body (Fig. 9A, B). Genetically, the closest relative of *G. irregularis* is *G. oculolineatus* (Duchene *et al*. 2013). In fact, the two species have a genetic distance of < 2%, which suggests intra- rather than interspecific variation. However, their colour pattern is distinct, in that *G. oculolineatus* has only two bars across the eye and suborbital area, similar to the first two in *G. irregularis*, but with a wide, conspicuously coloured (dark red to brown) interspace. In addition, *G. oculolineatus* has no lines on the body, not even as juveniles. Therefore, we consider the two species as distinct, similar to other closely related species pairs, such as *G*. sp. D sensu Munday *et al*. (1999) and *G. quinquestrigatus* (P. Munday, personal communication). *Gobiodon irregularis* is also a close relative of *G. reticulatus* Playfair 1867 (Fig. 11). Similarities with the latter exist in fin-ray counts but also in the head colour pattern of subadult *G*. *irregularis*. The two species can be discriminated not only genetically but also based on life colouration in the field: *G. reticulatus* has large spots on the body and 5 or 6 rather regular and broad bluish head bars, whereas the small spots in juvenile and subadult *G. irregularis* are restricted to the upper half of the body and adults have only two less distinct orbital bars. They also differ in their habitat requirements in that *G. reticulatus* usually lives in deep water (up to 30 m) and only occupies a few, particular *Acropora* species which are rarely used by other species (see Herler & Hilgers (2005) and Dirnwoeber & Herler (2007)). Another species with bright lines on the head as in *G. bilineatus*, *G. irregularis* and *G. oculolineatus* is *G. fulvus* sensu Winterbottom & Emery (1986). This species, however, is rarely referred to in the literature and the name *G. fulvus* seems commonly applied to a species which does not agree with Herre’s (1927) original description (he mentioned a black opercular spot, no headlines and a pale orange body, all of which contradict the species recorded by Winterbottom and Emery (1986) and the specimens from the Maldives, Japan and Taiwan examined herein). However, *G. fulvus* sensu Winterbottom & Emery (1986) can be distinguished from the three species mentioned above by a very dark body, two distinct bright blue lines with black borders across the eye that extend obliquely to the posterior edge of the preopercle, and distinct white bands along the D2 and A base (also with black borders). The fin-base bands may be hardly visible in life (JH, personal observation) and therefore this species may be most easily mistaken in the field with the species mentioned above.

### *Gobiodon ater*, sp. nov.

Black Coralgoby

**Figure 5:**
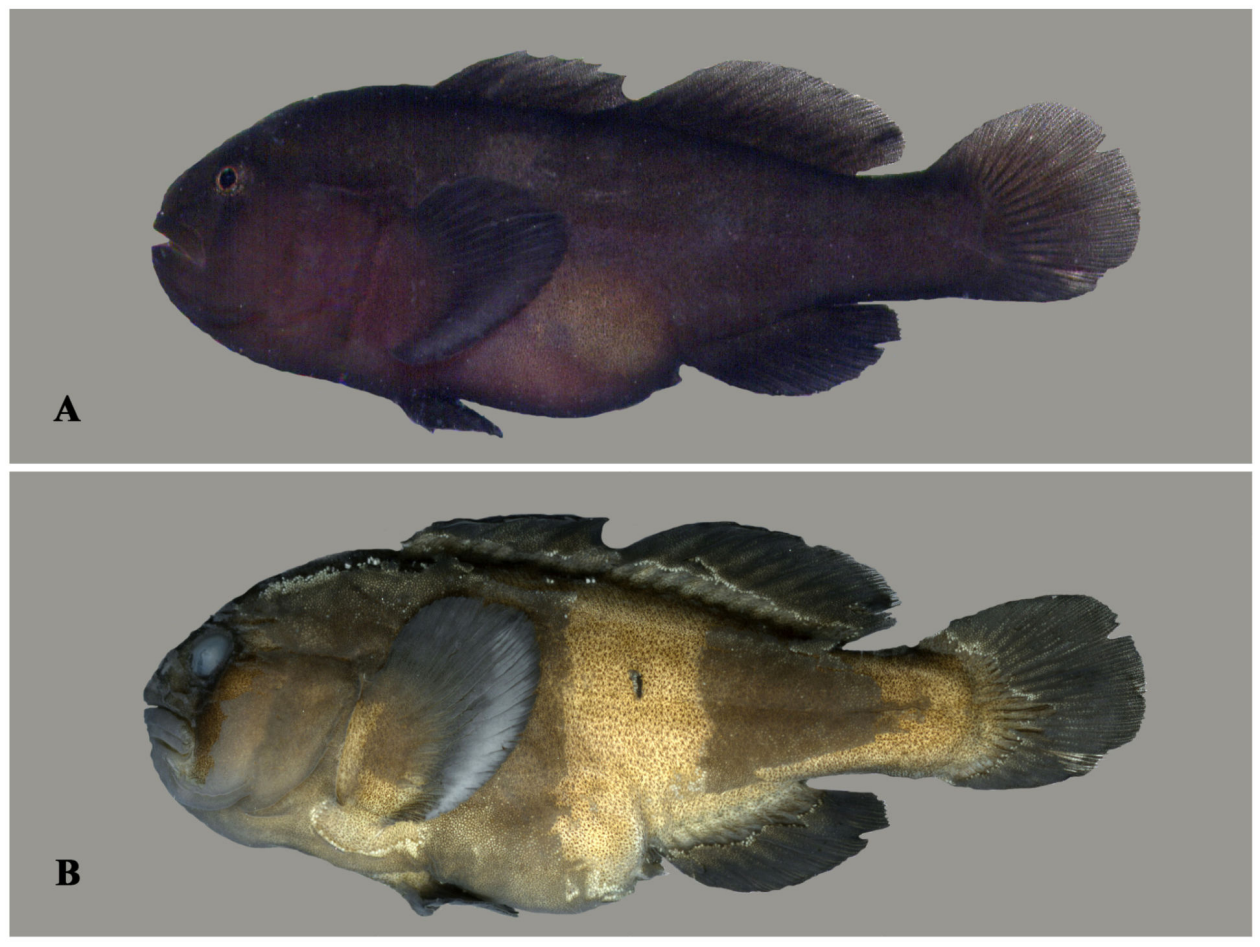
*Gobiodon ater* sp. nov., adult, holotype NMW 94612, 26.5 mm SL, Dahab, Egypt, Red Sea. **A.** freshly collected; **B.** ethanol-preserved (epidermal mucus partly removed). Photos by J. Herler.

**Figure 6:**
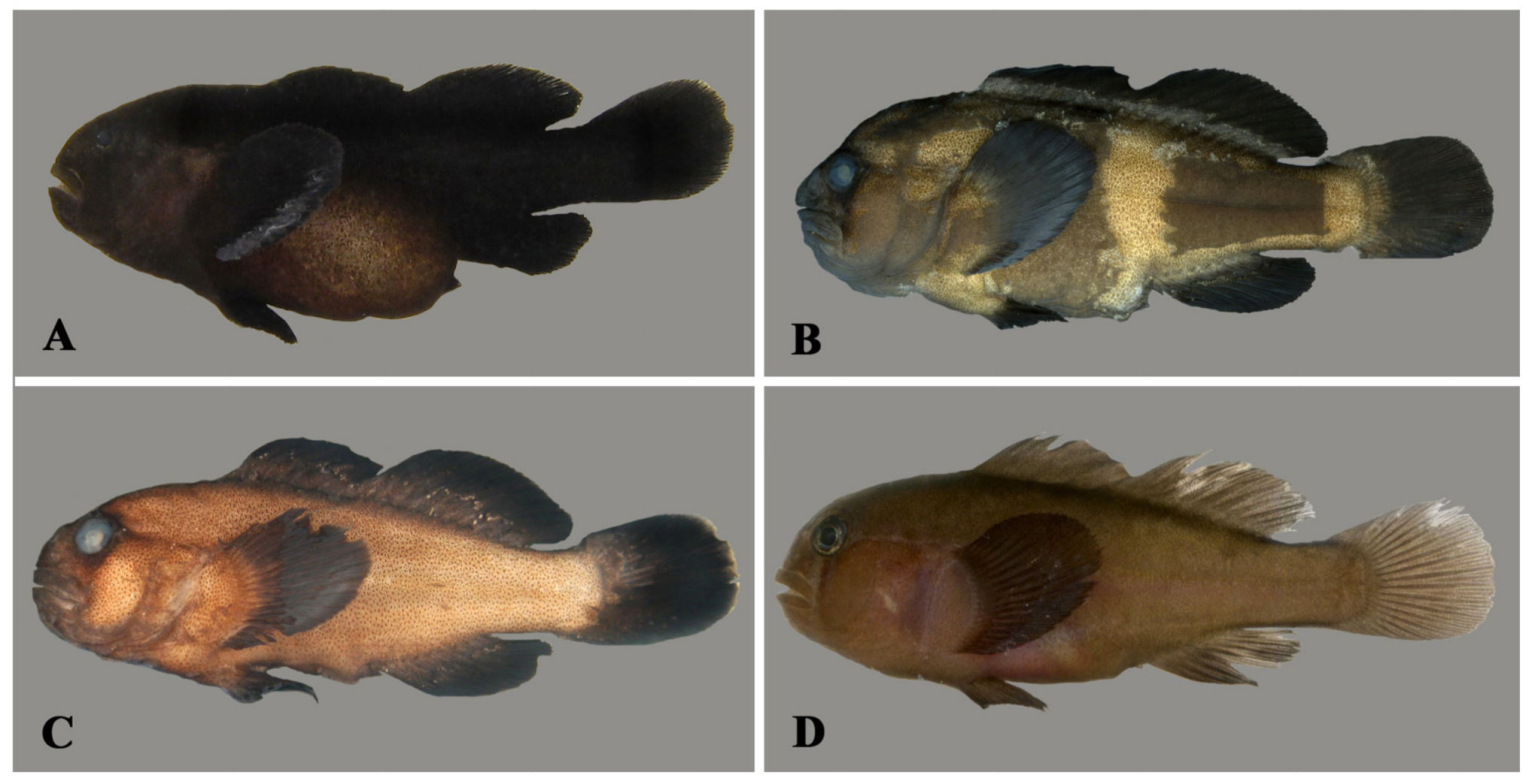
*Gobiodon ater* sp. nov. **A.** freshly collected, highly gravid female, CH 232-42-003, 18.5 mm SL, Dahab, Egypt, Red Sea; **B.** ethanol-preserved adult (epidermal mucus partly removed), paratype BMNH 2012.3.20.2, 24.6 mm SL, Dahab, Egypt, Red Sea; **C.** ethanol-preserved adult (epidermal mucus removed), CH 232-42-001, 18.2 mm SL, Dahab, Egypt, Red Sea; **D.** freshly collected adult, CH Mal 243, 19.1 mm SL, Gulhi Island, Maldives, Indian Ocean. Photos by J. Herler.

Figs. 5, 6, 10 and 11; Tables 1, 6, 7 and 10

*Gobiodon* sp. 2 Herler and Hilgers 2005: 121, Fig. 14; Niedermüller *et al*. (2009): 1501, Fig. 2; Herler *et al*. (2009): 733, Fig. 4.

#### Holotype

NMW 94612, female, 26.5 mm SL, Gulf of Aqaba, Egypt, Dahab, “Napoleon Reef” (28°28′15″ N, 34°30′33″ E), 1 m, coll. J. Herler, 18 November 2005.

#### Paratypes

Five specimens. NMW 94613, female, 26.4 mm SL, 14 November 2005, other data same as holotype. BMNH 2012.3.20.1, male, 23.1 mm SL, other data same as NMW 94613. BMNH 2012.3.20.2, female, 24.6 mm SL, other data same as NMW 94613. MNHN 2012-0110, female, 25.4 mm SL, other data same as holotype. MNHN 2012-0111, male, 15.9 mm SL, northern Red Sea, Egypt, Marsa Alam (25°03′59″ N, 34°54′05″ E), 1 m, coll. J. Herler, 29 November 2005.

#### Additional material

CH 232-42-001, 18.2 mm SL, Egypt, Dahab, coll. J. Herler, 30 November 2003; CH 232-42-003, 18.5 mm SL, Egypt, Dahab, coll. J. Herler, 26 May 2004; CH Mal 243 (DNA sample and photograph), 19.1 mm SL, Indian Ocean, Maldives, Gulhi Island, coll. J. Herler, 27 March 2007; CH Tai 007 (DNA sample), 18.2 mm SL, southern Taiwan, Kenting, coll. J. Herler, 04 December 2008.

#### Comparative material

*Gobiodon ceramensis* (Bleeker, 1853): OMNH P34042 (Fig. 9H), 35 mm SL, Japan, Ryukyu Islands, Ishigaki Island, coll. T. Suzuki and M. Suzuki, 17 August 1997.

#### Diagnosis

Dorsal-fin rays VI + I,10; anal-fin rays I,8; head and body naked; deep curved groove on isthmus; body and caudal peduncle slender (depth 37-41.2% and 13.8-14.7% SL, respectively); head rounded in juveniles, large adults slightly hump-headed. Juveniles and adults uniformly black, including iris. Very small species (< 28 mm SL); females of breeding pairs usually significantly larger than males, smallest highly gravid female 18.5 mm SL.

#### Description

(based on 6 types and several non-types (for osteology)). Large, compressed head; body rather cylindrical. Body proportions and meristics for types are provided in Tables 6 and 7, respectively. Unusual for the genus, the sexes differ in size: females of breeding pairs are usually significantly larger than the males. Dorsal-fin rays VI + I,10 (n = 6); anal-fin rays I,8 (n = 6); pectoral-fin rays **19**–20 (19:5, 20:1); pelvic-fin rays I,5; caudal fin with 16–17 branched and segmented rays. First dorsal-fin short and rounded. Second dorsal- and anal-fin with long posterior rays, rhomboid in shape. Pelvic disc well-developed, with frenum between spines. No scales. Gill opening as wide as pectoral-fin base, ending ventrally in opposite of 1^st^ or 2^nd^ lower pectoral-fin ray. Gill rakers 0–1 + 6–7. Deep curved groove present between interopercle and isthmus. Mouth terminal, bending downwards posteriorly, upper jaw reaching to below anterior margin of orbit or mid-orbit. Upper lip more curved down than lower lip. A few rows of small, slender and recurved teeth in both jaws behind the outer row, which has 10 larger and slightly recurved teeth in upper and lower jaw. In lower jaw, one pair of large, postsymphysial canines on each side; one or both of these teeth often smaller or absent, probably due to tooth loss and replacement. Anterior and posterior nasal openings at the end of short tubes. Head sensory canals typical for *Gobiodon* (Winterbottom & Harold 2005), with anterior oculoscapular (pores NA (paired), AI, PI (unpaired), SO, AO and IT (paired)) and preopercular (three pores on each side) canals present.

#### Life colouration

Uniformly black, including iris; thus eye hardly visible in living fish in the field (Figs. 5A, 6A). When stressed, black colouration can turn into grey.

#### Preserved colouration

After formalin fixation and ethanol preservation, the general colour is dark brownish-grey to black, eyes opaque. Some patches on the cheek, pectoral and median fins may appear lighter (Fig. 5B, 6A, 6C).

#### Molecular genetics

The present analysis includes the sequence of the a: paratype MNHN 2012-0111 (as G.sp.2_RN1) from the northern Red Sea (see Table 1 for Genbank accession numbers). Derived from the present analysis, the genetically closest species are *G. axillaris* and *G. fuscoruber*. The *p*-distance between *G. ater* and *G. fuscoruber* in the present study ranges from 0.023 to 0.032 (Table 10), making up a genetic difference of more than 2%. *Gobiodon axillaris* seems genetically even closer to *G. ater*, but since the NJ-bootstrap value between the two was very low (21), this relationship is highly questionable and requires a more detailed genetic and morphological analysis.

#### Habitat

*Gobiodon ater* is highly specialized and prefers fine-branched corals such as *Acropora selago* and small tabulate colonies of *A. hyacinthus*. Less often, it also occurs in other *Acropora* species, such as *A. acuminata*, *A. samoensis*, *A. eurystoma* and *A. valida* (Dirnwoeber & Herler 2007). The species is found in shallow water regions such as protected reef flats and reef crests. Due to its black colouration it is sometimes difficult to detect this species within the dark interstices of narrow-branched *Acropora* colonies.

#### Distribution

So far this species is known from the Red Sea, the central Indian and the western Pacific Ocean.

#### Etymology

This species is named after its uniformly black life colouration (“ater” = Latin word for “black”), which even includes the eye. Suggested common name: Black Coralgoby.

#### Remarks

This species was designated as *Gobiodon* sp. 2 by Herler and Hilgers (2005) and these authors assumed that it may be identical with one of the other entirely black species from the western Pacific, in particular with *G. ceramensis*. The syntypes of *G. ceramensis* cannot be identified from the more than 100 specimens collected by Bleeker (Ronald de Ruiter, pers. comm.), but when compared with the original description of Bleeker (1853), *G. ceramensis* has a higher fin-ray A count (9–10 rays versus 8), a lower P count (17 versus 19–20) and larger eyes (25 versus 21% of head length) than *G. ater*. The fin meristics of Bleeker (1853) are corroborated by specimens from Japan, which are considered to represent *G. ceramensis* (Fig. 9H) by one of us (TS); these also have fin-ray counts of D2 I,10 and A I,9, the range recorded by Bleeker (1853). Furthermore, according to Harold *et al*. (2008), *G. ceramensis* does not have a deep curved groove on isthmus, as it is present in *G. ater*. Other nominal species with an entirely black colouration (at least when adult) include *G. heterospilos* Bleeker 1856, *G. spilophthalmus* Fowler 1944, *G. albolineatus* Smith 1959, *G. albofasciatus* Sawada and Arai 1972, *G. acicularis* Harold and Winterbottom 1995, *G. winterbottomi* Suzuki, Yanao and Senou 2012 and a potentially new species from the Maldives (Herler *et al*. 2009; as *G*. sp. 4). Apart from the possibility that some of the four former species may be synonymous with each other, most of these species represent a genetical clade (tested for *G. acicularis, G. ceramensis* and *G. spilophthalmus* by Harold *et al*. 2008; Herler *et al*. 2009; Duchene *et al*. 2013 and herein (Fig. 11)), which is distant from *G. ater* and its genetically closest relatives *G. fuscoruber* and *G. axillaris*. Morphologically, *G. ater* differs from most members of the other genetic clade in that it is entirely black already when juvenile, and by a rounded first dorsal fin, shaped by a short first fin spine. By contrast, the first dorsal spine is the longest in several species of the other genetic clade. This results in a rather rectangular or even triangular first dorsal fin shape. Also typical for *G. ater* is the presence of a groove between interopercle and isthmus: a comparison with other species having such a groove is given in the remarks section of *G. fuscoruber* sp. nov. below. *Gobiodon ater* is genetically closely related to *G. axillaris* (Fig. 11, Duchene *et al*. 2013), although they have very different life colouration, and to *G. fuscoruber* sp. nov, to which it is phenotypically more similar. These species are in a clade together with another undescribed black species from the Indian Ocean (*Gobiodon* sp. 4, Herler *et al*. 2009), which has a very elongate body, and with *G. histrio* and *G. erythrospilus* Bleeker 1875. Considerable morphological similarities between *G. ater* and *G. fuscoruber* sp. nov. have also been revealed by the geometric morphometric analysis, in which the former in particular overlaps with western Pacific specimens of the latter (Fig. 10). MANOVA, however, confirmed significant shape differences between all three populations. Species discrimination was also supported by the PCA of body proportions of the Red Sea type specimens (adults only): *G. ater* mainly differs from *G. fuscoruber* sp. nov. by its longer abdomen (V—A: 30.6–34.3 versus 25.0–29.8% SL) and lower posterior body depth (D2—A: 29.5–32.3 versus 33.6–37.2% SL). They can further be distinguished by life colour and maximum size. Inferred from this morphological comparison and from a previous, more elaborate genetic analysis (Herler *et al*. 2009) that included sequences of *cytochrome* b, it is evident that *G. ater* and *G. fuscoruber* sp. nov. are very closely related but distinct species. The genetic distance between the two species calculated herein exceeds the level of 2% accepted for species delineation (Harold *et al*. 2008). The genetic distance to *G. unicolor* sensu Harold *et al*. (2008) from the Great Barrier Reef, being synonymous with *G. fuscoruber* sp. nov. (see below for details), is even greater (0.032).

### *Gobiodon fuscoruber*, sp. nov.

Brown-red Coralgoby

**Figure 7:**
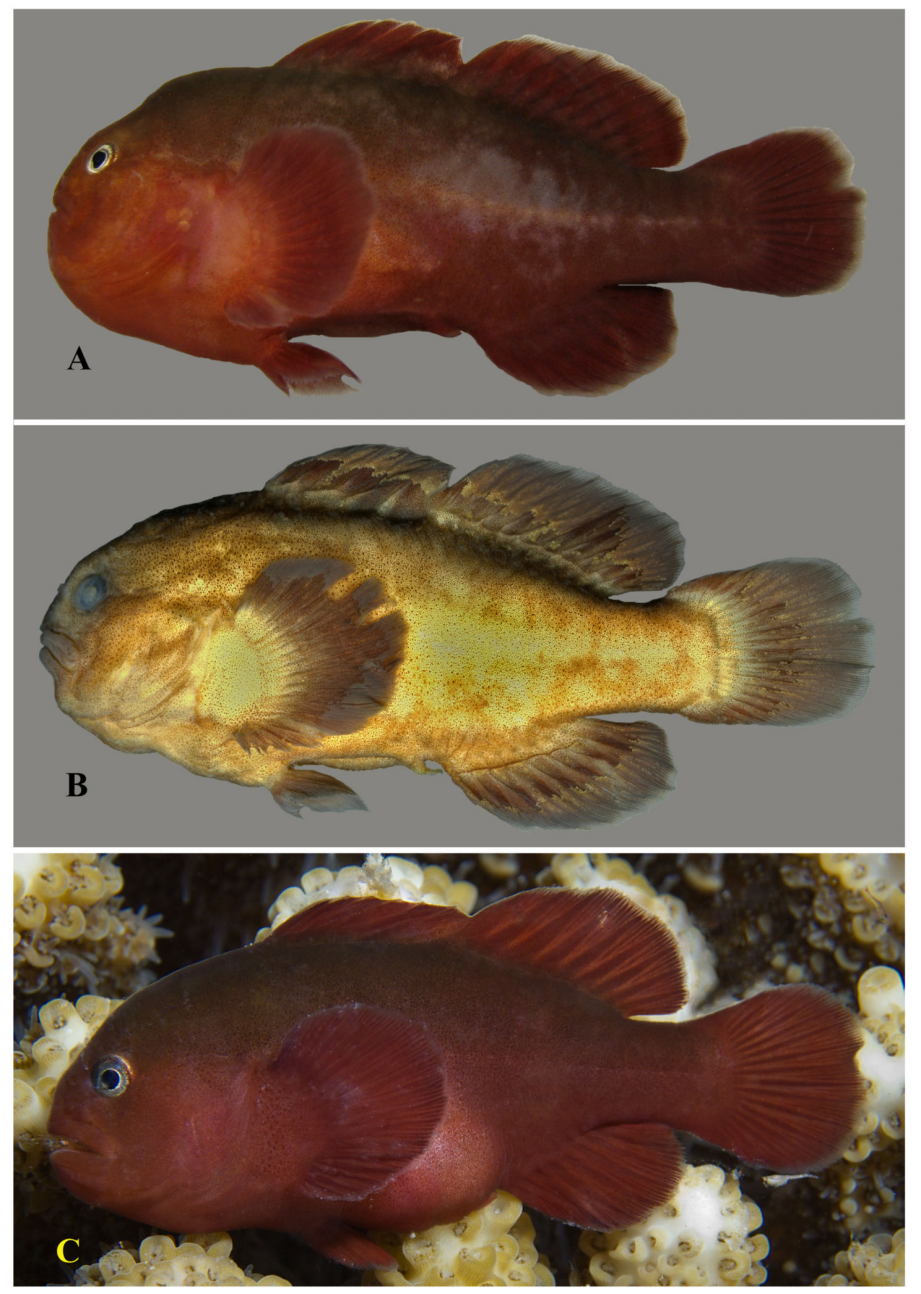
*Gobiodon fuscoruber* sp. nov. Holotype, NMW 95079, 36.7 mm SL, Dahab, Egypt, Red Sea. **A.** freshly collected, **B.** preserved; C: alive adult, PMR VP3202, 32 mm SL, Sharm el Moya, Egypt, Red Sea. Photos by J. Herler (A, B); S.V. Bogorodsky (C).

**Figure 8:**
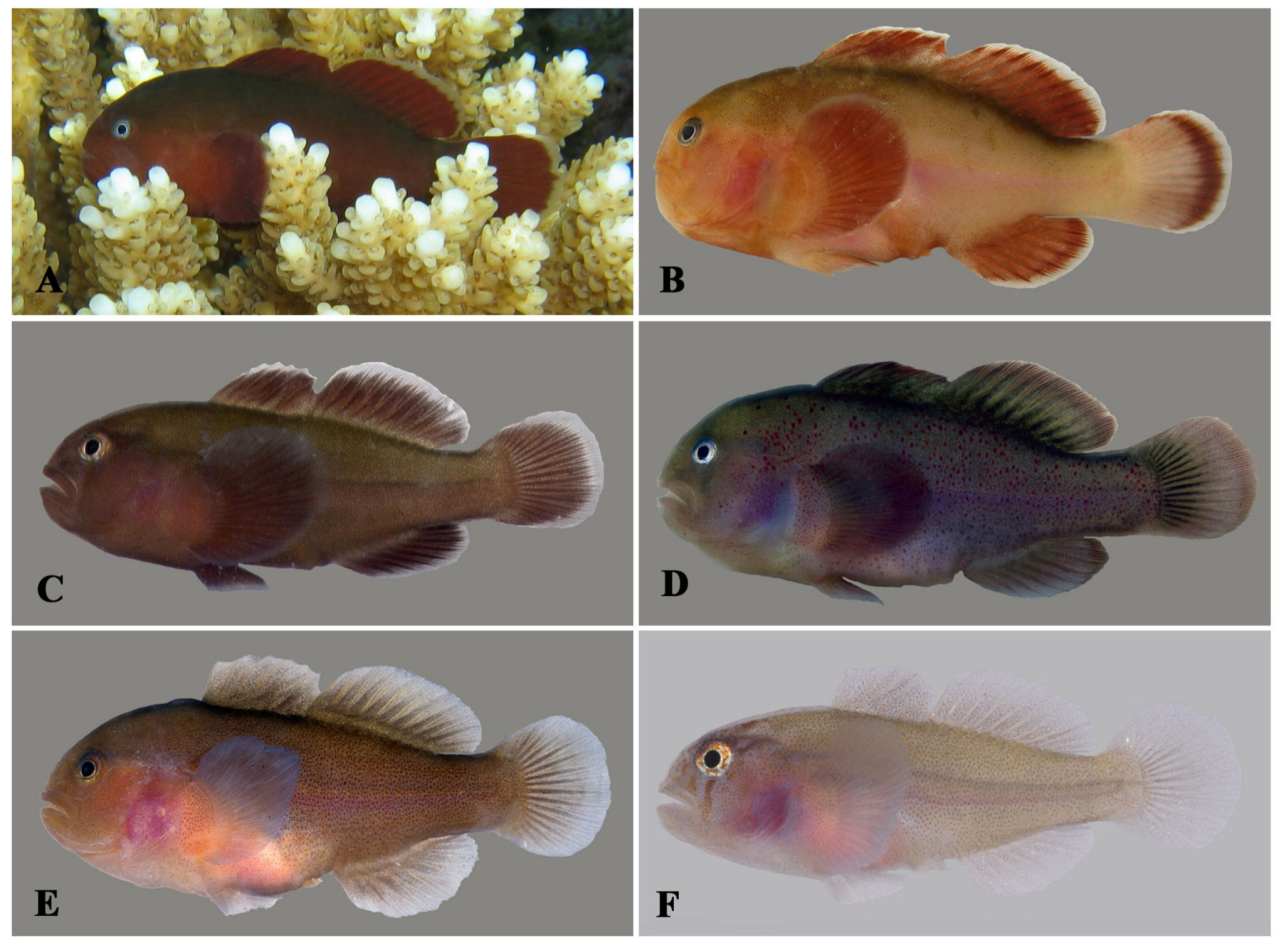
*Gobiodon fuscoruber* sp. nov. **A.** alive adult, holotype NMW 95079, 36.7 mm SL, Dahab, Egypt, Red Sea; **B.** freshly collected adult, CH 232-43-006, 31.5 mm SL, Dahab, Egypt, Red Sea; **C.** freshly collected juvenile, CH 232-43-015, 19.2 mm SL, Dahab, Egypt, Red Sea; **D.** freshly collected adult, CH 232-43-040, 26.2 mm SL, Fesdu Island, Maldives, Indian Ocean; **E.** freshly collected adult, paratype, OMNH 39990, 28.5 mm SL, Iriomote Island, Ryukyu Islands, Japan, western Pacific Ocean; **F.** freshly collected juvenile, OMNH 39978, 18.1 mm SL, Iriomote Island, Ryukyu Islands, Japan, western Pacific Ocean. Photos by J. Herler (A-D), T. Suzuki (E, F).

Figs. 7, 8, 10 and 11; Tables 1, 8, 9 and 10

*Gobiodon unicolor* (*non* Castelnau, 1873) Munday *et al*. (1999): 56, Fig. 13; Hayashi & Shiratori (2003): 63, Fig. 116; Senou *et al*. (2004): 170; Harold *et al*. (2008): 132.

*Gobiodon* sp. 5 Akihito *et al*. (2002): 1190.

*Gobiodon* sp. D Senou *et al*. (2004): 175.

*Gobiodon* sp. 3 Herler and Hilgers (2005): 123, Fig. 15; Niedermüller *et al*. (2009): 1501, Fig. 2; Herler *et al*. (2009): 733, Fig. 4.

#### Holotype

NMW 95079 (cited erroneously as CH 232-42-003 (correct number: CH 232-43-003) in Herler & Hilgers, 2005), male 36.7 mm SL, Gulf of Aqaba, Egypt, Dahab, “Islands” (28°28′38.50″ N, 34°30′47.10″ E), 1.5 m, coll. J. Herler, 27 March 2004.

#### Paratypes

Seven specimens. NMW 95080, 31.3 mm SL, 3 m, 11 November 2005, other data same as holotype. MNHN 2006–1700, 32.1 mm SL, 2 m, 17 November 2005, other data same as holotype. BMNH 1951.1.16.555, 29.2 mm SL and BMNH 1951.1.16.556, 34.0 mm SL, Red Sea, Saudi Arabia, Sanafir Island, coll. N. Marshall (Manihine Expedition), 1951. OMNH 39984, female, 29.2 mm SL, Japan, Ryukyu Islands, Iriomote Island, coll. T. Suzuki, 07 August 1993. OMNH 39986, male, 23.6 mm SL, Japan, Ryukyu Islands, Iriomote Island, coll. T. Suzuki, 22 August 1994. OMNH 39990, Japan, Ryukyu Islands, Iriomote Island, male, 28.5 mm SL, coll. T. Suzuki, 27 July 1997.

#### Additional material

CH 232-43-006, 31.5 mm SL, Egypt, Dahab, coll. J. Herler, 15 June 2004; CH 232-43-015, juvenile, 19.2 mm SL, Egypt, Dahab, coll. J. Herler, 14 November 2005; CH 232-43-040, 26.2 mm SL, Maldives, Fesdu Island, coll. J. Herler, 17 March 2007; PMR VP3202, 32 mm SL, Egypt, Sharm el Sheikh, Sharm el Moya, coll. S.V. Bogorodsky, 04 July 2011; OMNH 39978, juvenile, 18.1 mm SL, Japan, Ryukyu Islands, Iriomote Island, coll. T. Suzuki, 08 August 1996. OMNH P-40047 (DNA sample), 30.3 mm SL, Japan, Ryukyu Islands, Iriomote Island, coll. T. Suzuki, M. Suzuki and A. Kawai, 22 August 2002.

#### Comparative material

**Figure 9:**
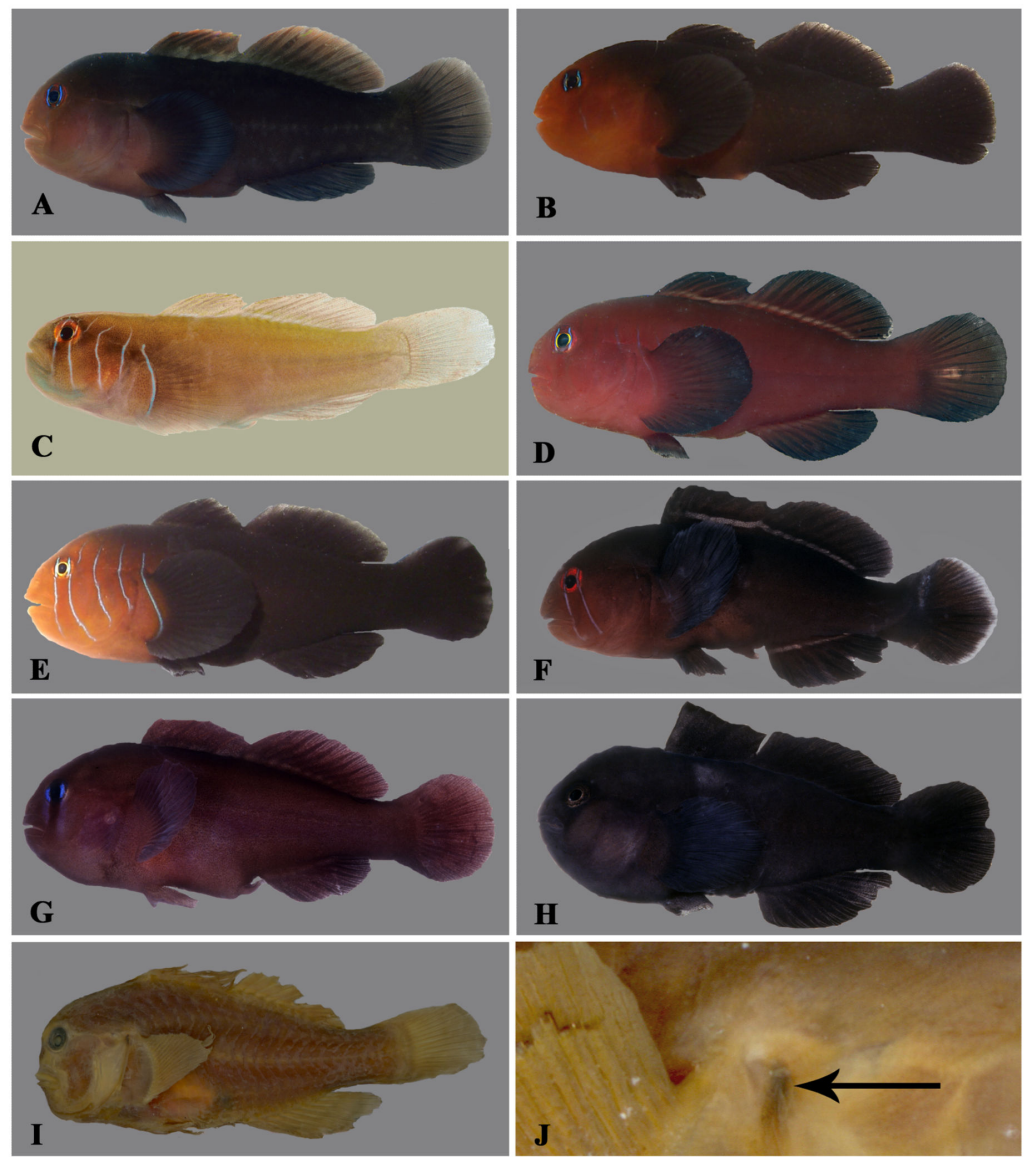
Comparative material. Freshly collected adults: *Gobiodon* cf. *bilineatus* sp. nov. **A.** uncatalogued, 28.9 mm SL, Kagi Island, Maldives. **B.** uncatalogued, 22.9 mm SL, Kenting, southern Taiwan. **C.** *Gobiodon prolixus*, ROM 73338, holotype, male 26.2 mm SL, Nha Trang, Vietnam. **D.** *Gobiodon* sp. D sensu Munday *et al*. (1999), uncatalogued, 29.9 mm SL, Hembadhu Island, Maldives **E.** *Gobiodon quinquestrigatus*, uncatalogued, 28.7 mm SL, Kenting, southern Taiwan; **F.** *Gobiodon* cf. *fulvus* (sensu Winterbottom & Emery 1986), OMNH P40259, 20.8 mm SL, Iriomote Island, Japan. **G.** *Gobiodon oculolineatus*, OMNH P40260, 22.6 mm SL, Okinawa Island, Japan. **H.** *Gobiodon ceramensis*, OMNH P34042, 35 mm SL, Ryukyu Island, Japan. *Gobiodon unicolor*, preserved holotype, MNHN A-4015, 31 mm SL, **I.** entire specimen. **J.** close-up of black opercular spot (arrow) on the right side. Photos by J. Herler (A, B, D, E, I, J), R.Winterbottom (C), T. Suzuki (F-H).

*Gobiodon unicolor* (Castelnau 1873): holotype, MNHN A-4015 (Fig. 9I, J), 29.6 mm SL, Australia, Cape Sidmouth, coll. Castelnau.

#### Diagnosis

Dorsal-fin rays I,10; anal-fin rays I,8; head and body naked; obvious groove between isthmus and interopercle; body deep (the depth at pelvic-fin origin 40.4–44.3 % SL) and strongly compressed; head rounded in juveniles, large adults with steep profile, slightly hump-headed; caudal peduncle relatively slender (depth 14.8-16.0% SL). Juveniles and adults dark reddish brown with greenish subcutaneous gleam on dorsal part of body; median fins plain (western Pacific Oceans) or with pale margin (Red Sea and Indian Ocean); iris plain light blue (Red Sea) or scattered with red-brown dots on the outer margin.

#### Description

(based on 5 types and several non-type specimens (for osteology)). Head and body strongly compressed. Body proportions and meristics for types are provided in Tables 8 and 9, respectively. Dorsal-fin rays VI + I,10 (n = 8); anal-fin rays I,8 (n = 8); pectoral-fin rays 19–20 (19:4, 20:4); pelvic-fin rays I,5, fin short (not reaching anus) and cup-shaped with significant frenum between spines; caudal fin with 15–17 segmented and branched rays. First dorsal fin lower than second dorsal and anal fins. Vertebral column with 10 precaudal and 16 caudal vertebrae, including urostyle. No scales. Gill opening as wide as pectoral-fin base, ending ventrally in opposite of 1^st^ or 2^nd^ lower pectoral-fin ray. Gill rakers 0–2 + 7–8. Obvious groove between interopercle and isthmus. Mouth slightly oblique, bending downwards and reaching approximately to below anterior margin of orbit. Upper lip usually slightly curved, slightly extending before snout. One outer row of 3 to 8 larger, slightly recurved teeth in upper and lower jaw, positioned on the anterior half of premaxilla and dentary. Medially, 3 to 4 rows of small, slender and recurved teeth in both jaws. In lower jaw, a pair of large, postsymphysial canines on each side, one or both on each side often small or absent, probably due to tooth loss and replacement. Anterior and posterior nasal openings at the end of short tubes. Head sensory canals typical as for *Gobiodon* (Winterbottom & Harold 2005), with anterior oculoscapular (pores NA (paired)), AI, PI (unpaired), SO, AO and IT (paired) and preopercular (three pores on each side) canals present.

#### Life colouration

Body uniformly dark reddish brown, occasionally with a greenish gleam on dorsal part of body (in Red Sea specimens), densely dotted with dark brown on body, nape, and pectoral-fin base (Figs. 7A, C, 8); specimens from the Maldives characterised in having scattered tiny red spots; a weak purple streak of internal pigment runs along the lateral midline of the body, from behind the pectoral fins to the caudal-fin origin; iris plain light blue or scattered with red-brown dots on the outer margin; median fins with pale margin typically in specimens from the Red Sea and sometimes in specimens from the Maldives but fins plain in specimens from western Pacific Ocean.

#### Preserved colouration

Head and body uniformly dark brown. When mucous epidermis removed, body light brown with numerous small dark chromatophores scattered all over (Fig. 7B). Iris bright. Pale margins on median fins often retained.

#### Molecular genetics

In the analysis here, the sequences of G.sp3_MA1 from the Maldives and of G.sp3_GA1 from the Gulf of Aqaba, Red Sea by Herler *et al*. (2009) are included. See Table 1 for Genbank accession numbers. *Gobiodon fuscoruber* sp. nov. is closely related to *G. ater* sp. nov. (see above). The intraspecific *p*-distances between populations from the Red Sea, the Maldives, Japan and the GBR (as “*G. unicolor*”) are around 0.02 (see Table 10), with the least distance between the two latter (0.001).

#### Habitat

On reef flats, crests and upper reef slopes in less exposed areas. Prefers *Acropora selago* but also occurs in a number of other, narrowly branched *Acropora* species such as *A. acuminata* and *A. hyacinthus*.

#### Distribution

The species is currently known from the Red Sea, central Indian Ocean and western Pacific Ocean (Japan and GBR).

#### Etymology

This species is named after its uniformly reddish-brown life colouration. The name “*fuscoruber*” is a combination of the Latin words “*fuscus*” (= brown) and “*rubrum*” (= red). Although there are geographic colour variants (e.g. red dots in the Maldives), the reddish brown basic colouration is typical throughout its distribution area. Suggested common name: Brown-red Coralgoby.

#### Remarks

This species was informally designated as *Gobiodon* sp. 3 by Herler and Hilgers (2005). The Red Sea specimens are very similar to populations from the Maldives and the western Pacific, especially in body shape, meristics (D2 I/10, A I/8 in all specimens from the Maldives and the western Pacific) and basic colour pattern. The Maldivian specimens, however, are somewhat brighter, the blue iris less obvious, and they have red dots on the postorbital area, nape, and body (more densely on the dorsum). They are similar to the Red Sea population in having reddish brown median fins, sometimes also with a pale margin. Specimens from Japan have a somewhat brighter body colouration (especially in juveniles) and the scattered dark brown chromatophores are more obvious than in the usually dark reddish brown Red Sea specimens. In addition, Japanese specimens often have two weak red bars below the orbit and several short red lines and dots on the anterior nape and snout. Suborbital bars may be retained in subadults but are very rarely seen in adults. Despite some geographic variation in colouration, the genetic distance of the populations investigated is very small. Therefore, we assume that these populations belong to one species.

*Gobiodon fuscoruber* was assumed to be identical with *G. unicolor* (Castelnau 1873) after comparison with images in Munday *et al.* (1999) and Senou *et al*. (2004). Genetic investigations confirm that *G. unicolor* sensu Munday *et al*. (1999) and Harold *et al*. (2008) is identical with *G. fuscoruber* (Fig. 11, Table 10). Examinations of the holotype of *G. unicolor*, however, revealed that this specimen represents a species with a black opercular spot (see Figs. 9I, J), which is characteristic for several species of the genus, but is present neither in *G. fuscoruber* nor in *G. unicolor* noted by any of the authors mentioned above. Considering the black opercular spot and the general body shape of the holotype of *G. unicolor*, the type species could be identical with *G. histrio, G. axillaris* or *G.* sp. C of Munday *et al*. (1999), which all have a black opercular spot. When comparing body shapes in detail, it becomes clear that the holotype represents *G. histrio* (Valenciennes 1837). This is confirmed by a comparison of the two preserved syntypes (MNHN 3098) and x-ray images of *G. histrio* with the preserved holotype and x-ray image of *G. unicolor*: the types of both species resemble each other in that they have a deep and compressed body, a groove between the interopercle and isthmus, strongly re-curved jaws and identical fin ray counts in D2 and A (10 and 9, respectively; except for one syntype of *G. histrio* that has 11 D2-rays). The colour description of *G. histrio* by Cuvier and Valenciennes (1837) and the range of fin rays in D2 and A of the syntypes confirm its identity with *G. histrio* shown by recent authors (e.g., Munday *et al*. 1999, Herler & Hilgers 2005). Further evidence of the synonymy of *G. histrio* and *G. unicolor* comes from Castelnau’s (1873) description of the preserved colouration of *G. unicolor* as a light brown. When specimens of *G. histrio* are preserved, the typical green-red colouration fades very quickly after ethanol preservation and also turns uniformly light brown (see Herler & Hilgers 2005).

**Figure 10:**
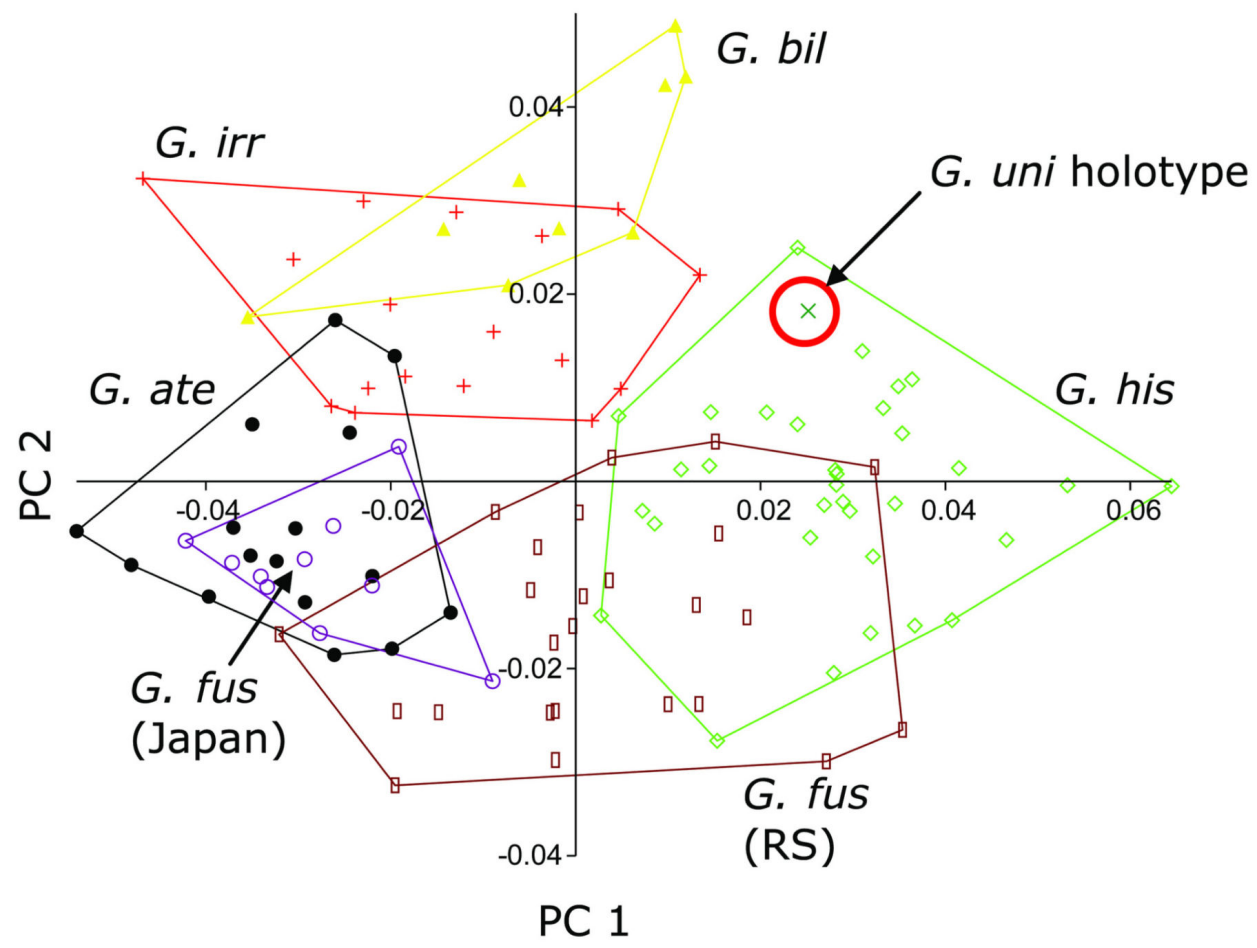
First two principal components of a PCA of Procrustes coordinates of 15 landmarks and 6 semi-landmarks put on 111 specimens of *Gobiodon*. The holotype (MNHN A-4015) of *G. unicolor* (Castelnau, 1873) is marked by a red circle. RS = Red Sea. For species abbreviations see Table 1.

**Figure 11:**
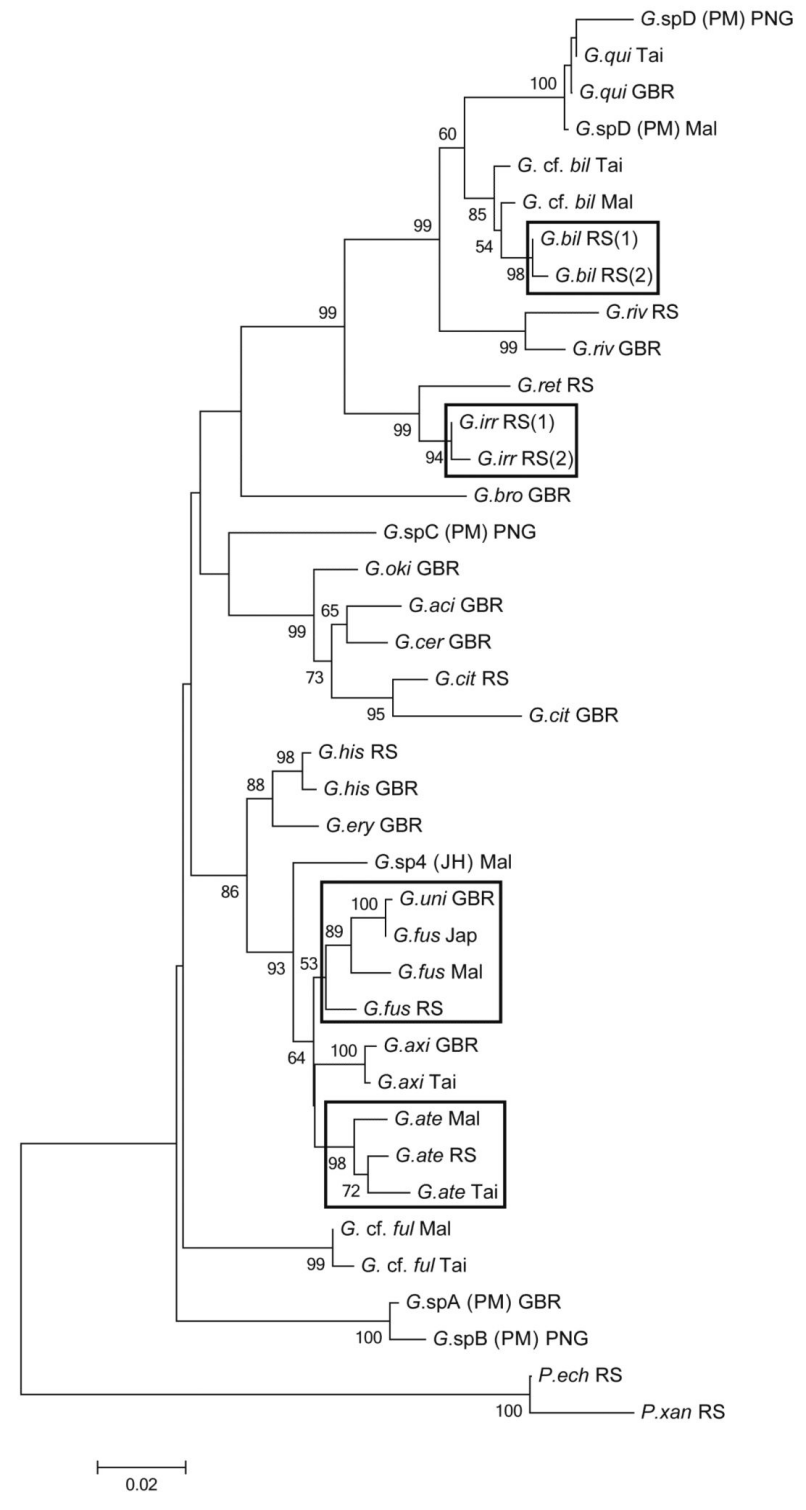
Neighbor-joining tree of 23 taxa of *Gobiodon* and 2 *Paragobiodon* species (as nearest neighbour at genus level) based on combined sequences of partial 12S and 16S rRNA mitochondrial genes. According to a model test in MEGA 5.05, a K2P-model with a gamma-parameter of 0.23 and pairwise deletions of gaps/missing data were selected. Numbers at nodes are bootstrap values (1000 replicates); values <50 are not shown. Bar below tree shows substitutions per site. The 4 new species are marked with a box. For abbreviations of species and localities see Table 1. Undescribed species are given with references in parentheses: JH = according to Herler *et al*. (2009), PM = according to Munday *et al*. (1999).

The final confirmation of the synonymy of *G. unicolor* and *G. histrio* comes from a geometric morphometric analysis. Principal component analysis plotted the holotype of *G. unicolor* clearly within the morphospace of *G. histrio* (Fig. 10), and discriminant function analysis on the scores of the first 6 PCs of all 111 specimens revealed a 99.99% probability of assignment to *G. histrio*. We therefore propose *G. unicolor* (Castelnau 1873) to be a junior synonym of *G. histrio* (Valenciennes 1837) and as not applicable to the uniformly coloured species described herein. The new name *G. fuscoruber* not only has to be used for the species called *G. unicolor* by several authors mentioned above, but also applies to some undescribed species, such as *G*. sp. 5 of Akihito *et al.* (2002), and *G*. sp. D of Senou *et al*. (2004). Apart from genetics (at least tested for “*G. unicolor*”), the uniformly (brownish) life colour, the rounded head shape, the low first dorsal fin and the large pectoral fins unite these taxa. This suggests that *G. fuscoruber* is a widely distributed species, occurring throughout the Indo-Pacific reef province.

*Gobiodon fuscoruber* and *G. ater* share a special feature with at least seven other species of the genus: a groove between the interopercle and isthmus (Harold & Winterbottom 1999; Harold *et al*. 2008), which is also present in *G. brochus* Harold and Winterbottom 1999 and *G. flavus* Sauvage 1880. However, these species are clearly distinct from *G. ater* and *G. fuscoruber* in that they have a much brighter colouration. Furthermore, *G. flavus* and *G. brochus* have at least 9 anal fin rays (Sauvage 1880, Harold & Winterbottom 1999) instead of the very constant number of 8 rays in *G. fuscoruber* and *G. ater*. Moreover, the colour description of Sauvage (1880) for *G. flavus* notes a yellowish colouration. The combination of this colouration and presence of the groove makes assignment of *G. flavus* to any of the currently known species difficult, though it is reasonable to conclude that it is not identical with any of the species described herein. Other species with such a groove, like *G*. sp. A, sp. B, sp. C, *G. histrio* and *G. erythrospilus* (Harold *et al*. 2008) are very different in their life coloration and cannot be mistaken with *G. fuscoruber* or *G. ater*.

## Discussion

The four new species—*G. bilineatus, G. irregularis, G. ater* and *G. fuscoruber*—are morphologically and genetically distinct species. Apart from several nominal species described in the past and discussed above, there are some more species of *Gobiodon* which have been formally described, but which are difficult to assign to any of the presently known species as a consequence of inadequate original descriptions, a lack of illustrations and/or the absence of clearly designated types. However, comparisons with original descriptions, and, when available, type material, have proven that for example neither *G. coryphaenula* (Valenciennes 1837) nor *G. erythrophaios* (Bleeker 1849) is identical with any of the four species described herein. The holotype of *G. coryphaenula* is distinct from the new species described herein by a well-recognisable black opercular spot (examination by JH), and by a low D2 and A count (9 and 8 rays, respectively), as noted by Cuvier and Valenciennes (1837). Potential conformities with *G. erythrophaios* are more difficult to clarify, because the types are not really known and the material by Bleeker stored in the Dutch Natural History Museum in Leiden contains 103 specimens, which may even include the types of *G. ceramensis* (Ronald de Ruiter, personal communication). However, apart from the low pectoral count (17 rays) of *G. erythrophaios* given by Bleeker (1849), the coloration in the original description notes a red head without any lines, and a brown body. Considering all these features, none of the four present species resembles the type description of Bleeker (1849). However, *G. erythrophaios* may be similar to or identical with specimens from the Indian/western Pacific Ocean, designated as *G*. cf. *bilineatus* herein, at least when referring to the life colouration.

In the case of *G. fuscoruber*, we have shown that it is identical with a well-known Indian Ocean/western Pacific species that has been erroneously called *G. unicolor* in the past (see for example Munday *et al*. 1999, Senou *et al*. 2004, Harold *et al*. 2008). This assumption has now also been proven genetically. All populations of *G. fuscoruber* had a genetic distance < 2% and the populations from Japan and the GBR (“*G. unicolor*”) are almost identical. As distinct morphological features, such as basic life colouration, meristics and morphometrics, are congruent across regions, we refrained from designating subspecies, and assume that populations at such great distances can easily develop deviations in their colour pattern. Although *G. fuscoruber* is distinct from *G. ater*, a more detailed analysis by Herler *et al*. (2009), including a third molecular marker, showed that the Indian Ocean *G. fuscoruber* is similar to *G. ater* from the Red Sea, but relatively far from *G. fuscoruber* in the Red Sea. This is also reflected in the present geometric morphometric analysis, in which Japanese specimens of *G. fuscoruber* are closer to *G. ater* than to their Red Sea conspecifics. We therefore assume that *G. ater* and *G. fuscoruber* have diverged in the Indian or western Pacific Ocean and that the Red Sea population of the latter may have been long isolated from its eastern counterpart. It has also been shown that geometric morphometrics is a useful tool even for matching recent specimens with very old types, in our case confirming that *G. unicolor* (Castelnau 1873) is a junior synonym of *G. histrio* (Valenciennes 1837).

Geographic distance causes distinctness, both morphologically and genetically, even in such conservative genes as 12S and 16S. In some species (*G. bilineatus, G. ater*, and *G. fuscoruber*), representatives from more than two regions were sequenced, and, with the exception of *G. ater*, geographic distance was correlated with genetic distance. Although the small sample size and the conservative genetic markers do not permit extensive phylogeographic discussions, we were able to delimit species despite intraspecific geographic variation. We show that large geographic distances (several 1000 km) between populations may result in within-species genetic distances that are comparable to or even larger than those of sympatric species. Although a genetic distance of about 2% seems feasible for species delimitation in several cases, this of course depends on the phylogenetic history, population sizes and on the larval distribution patterns of species. Harold *et al*. (2008), for example, found genetic differences of far less than 2% in morphologically distinct species such as *Gobiodon* sp. A and sp. B. Similar observations were made for the very closely related *G. irregularis* and *G. oculolineatus*. In addition, the two taxa *Gobiodon* sp. D sensu Munday *et al.* (1999) and Harold *et al.* (2008) and *G. quinquestrigatus* are considered to be distinct species (Duchene *et al*. 2013), although they differ little genetically (1.1–1.3%). Moreover, the identity of the true *G. quinquestrigatus* remains to be clarified because the description of the life coloration of *G. quinquestrigatus* by Cuvier and Valenciennes (1837) (reddish brown basic coloration, and darker, brown fins) resembles *G*. sp. D rather than the dark-bodied species commonly referred to as *G. quinquestrigatus* (see above for details). The close genetic relationship of several distinct species suggests that recent speciation events have occurred in the genus *Gobiodon* as it was confirmed in a recent phylogenetic analysis (Duchene *et al*. 2013). In contrast, our analysis revealed a within-species genetic distance of 2.3% between Red Sea and western Pacific *Gobiodon rivulatus* and even more than 3% in *Gobiodon citrinus*. There is little doubt that these distant populations represent the same species morphologically (i.e., there is little or no variation in life colouration across this range), and higher genetic distances are not surprising in such widely distributed species with very large populations.

The present study, in combination with the species noted by Herler and Hilgers (2005), has shown that the Red Sea is rich in coral-associated gobies of the genus *Gobiodon*: nine species are now recorded (including the recent discovery of *G. prolixus* by Bogorodsky *et al*. (2010)). Overall, the genus *Gobiodon* may be much more diverse than previously assumed. On the one hand, new species have been discovered recently and on the other hand, several known species are still undescribed (Munday *et al*. 1999, Senou *et al*. 2004). Our present NJ-analysis includes 23 taxa, which may represent a lower number of true species, but lacks at least 9 described and probably valid species (G. albolineatus, G. albofasciatus, G. heterospilos, G. micropus Günther 1861, *G. multilineatus* Wu 1972, *G. prolixus, G. oculolineatus, G. spilophthalmus* and *G. winterbottomi*). Combining this information, and although several of these taxa require further taxonomic investigations, we are sure that more than 30 species of *Gobiodon* exist.

In summary, the combined investigation of simple morphological characters (such as life colouration and fin meristics), molecular data and multivariate-statistical morphometric analyses is a feasible approach for species discrimination and delimitation within *Gobiodon* and may even allow making decisions about old and “difficult” type material.

## Key to species of Gobiodon from the Red Sea

**Table:** 

1a.	Head and body all plain black; caudal-peduncle depth 14–15% SL	*G. ater* sp. nov.
1b.	Colour not as above; caudal-peduncle depth 15–18% SL	2
2a.	Black or dark blue spot (usually retained when preserved) at upper posterior margin of operculum	3
2b.	No dark spot at upper corner of operculum	4
3a.	Body depth 38–42% SL; no median groove on isthmus; yellow in life, with two blue lines through eye, and two blue lines before and behind opercular spot	*G. citrinus*
3b.	Body depth 42–48% SL; a median groove on isthmus, widening posteriorly; green to bluish green, with four orange-red bars on side of head dividing dorsally, and with longitudinal, vermiculate, orange-red bands dorsally on body	*G. histrio*
4a.	Many, slightly oblique, light blue lines on body behind pectoral-fin base, lines becoming more irregular posteriorly	*G. rivulatus*
4b.	No vertical lines on body	5
5a.	Body deep, depth 40-44% SL; colour uniformly dark reddish brown, densely dotted with dark brown on body, nape, and pectoral-fin base; no bars or lines on the head; iris light blue	*G. fuscoruber* sp. nov.
5b.	Body slender or moderately deep, depth 28–40% SL; head with vertical red or blue bars or lines, or two or three bars through the eye	6
6a.	Body brownish orange to red, with numerous, small pale blue spots; five vertical bluish bars on head and two on pectoral-fin base; base of dorsal and anal fins with a dark reddish- or black-edged pale blue band, margin of the fins often yellow	*G. reticulatus*
6b.	No spots on body or irregular red spots and lines only on upper half of body (juveniles and subadults of *G. irregularis*); head with vertical blue lines or red bars below the eye; base of dorsal and anal fins without band (or with a narrow bluish line in some subadults of *G. bilineatus*)	7
7a.	Uniformly reddish brown (adults) or with red spots and irregular short lines on upper half of head and body (juveniles and sub-adults); head with three orange-red bars below eye, indistinct in adults	*G. irregularis* sp. nov.
7b.	No spots on head and body; head with five blue lines or at least two blue lines across eye (adults of *G. bilineatus*)	8
8a.	Adults and juveniles translucent greenish grey; five vertical blue lines on the head and pectoral-fin base in juveniles and adults; ovate internal bluish grey spots along vertebral column; body depth 28–36% SL; distance between anterior D1 insertion and dorsal insertion of pectoral-fin 42–50% of head length	*G. prolixus*
8b.	Adults and subadults uniformly bright orange-red or dark red, juveniles greenish; no lines on the head (adults) or five blue vertical lines on the head and pectoral-fin base (juveniles and subadults); no internal spots visible along the vertebral column; body depth 36–40% SL; distance between D1 insertion and dorsal insertion of pectoral-fin 53–71% of head length	*G. bilineatus* sp. nov.

## Figures and Tables

**Table 1:** GenBank accession numbers (GB No.) and references for published sequences (12S and 16S rRNA) of 25 taxa (4 new species are underlined) of *Gobiodon (G.)* and *Paragobiodon (P.)*. Localities: GBR = Great Barrier Reef, Mal = Maldives, PNG = Papua New Guinea, RS = Red Sea, Tai = Taiwan. Abbreviations (PM) and (JH) refer to the first demonstration of sequenced but undescribed species, Munday *et al*. (1999) and Herler and Hilgers (2009), respectively. Superscript numbers in locality code indicate type status: a: Paratype MNHN 2012-0111, b: Paratype BMNH 2006.10.6.1, c: Paratype NMW 95565.

Nr.	Species	Abbrev.	Locality	GB No. 12S/16S	Sequ. Ref.
1	*G. acicularis*	G. aci	GBR	EF540565 EF463071	Harold *et al*. (2008)
2	*G. ater*	G. ate	RS(1)^a^	FJ617046 FJ617086	Herler *et al*. (2009)
	*G. ater*	G. ate	Mal	KF242349 KF242362	this study
	*G. ater*	G. ate	Tai	KF242350 KF242363	this study
3	*G. axillaris*	G. axi	GBR	EF540567 EF463074	Harold *et al*. (2008)
	*G. axillaris*	G. axi	Tai	KF242351 KF242364	this study
4	*G. bilineatus*	G. bil	RS (1)^b^	KF242352 KF242365	this study
	*G. bilineatus*	G. bil	RS (2)	KF242353 KF242366	this study
5	*G. cf. bilineatus*	G. cf. bil	Mal	KF242354 KF242367	this study
	*G. cf. bilineatus*	G. cf. bil	Tai	KF242355 KF242368	this study
6	*G. brochus*	G. bro	GBR	EF540565 EF463071	Harold *et al*. (2008)
7	*G. ceramensis*	G. cer	GBR	EF540570 EF527238	Harold *et al*. (2008)
8	*G. citrinus*	G. cit	GBR	EF540572 EF527240	Harold *et al*. (2008)
	*G. citrinus*	G. cit	RS	FJ617027 FJ617067	Herler *et al*. (2009)
9	*G. erythrospilus*	G. ery	GBR	EF540574 EF527242	Harold *et al*. (2008)
10	*G. cf. fulvus*	G. cf. ful	Mal	KF242356 KF242369	this study
	*G. cf. fulvus*	G. cf. ful	Tai	KF242357 KF242370	this study
11	*G. fuscoruber*	G. fus	RS	FJ617047 FJ617087	Herler *et al*. (2009)
	*G. fuscoruber*	G. fus	Mal	FJ617048 FJ617088	Herler *et al*. (2009)
	*G. fuscoruber*	G. fus	Jap	KF242358 KF242371	this study
12	*G. histrio*	G. his	GBR	EF540576 EF527244	Harold *et al*. (2008)
	*G. histrio*	G. his	RS	FJ617031 FJ617071	Herler *et al*. (2009)
13	*G. irregularis*	G. irr	RS (1)	FJ617041 FJ617081	Herler *et al*. (2009)
	*G. irregularis*	G. irr	RS (2)^c^	KF242359 KF242372	this study
14	*G. okinawae*	G. oki	GBR	EF540578 EF527246	Harold *et al*. (2008)
15	*G. quinquestrigatus*	G. qui	GBR	EF540580 EF527249	Harold *et al*. (2008)
	*G. quinquestrigatus*	G. qui	Tai	KF242360 KF242373	this study
16	*G. reticulatus*	G. ret	RS	FJ617034 FJ617074	Herler *et al*. (2009)
17	*G. rivulatus*	G. riv	GBR	EF540581 EF527250	Harold *et al*. (2008)
	*G. rivulatus*	G. riv	RS	FJ617037 FJ617077	Herler *et al*. (2009)
18	*G.* species A (PM)*	G. spA	GBR	EF540560 EF443268	Harold *et al*. (2008)
19	*G.* species B (PM)	G. spB	PNG	EF540561 EF463067	Harold *et al*. (2008)
20	*G.* species C (PM)	G. spC	PNG	EF540563 EF463069	Harold *et al*. (2008)
21	*G.* species D (PM)	G. spD	PNG	EF540564 EF463070	Harold *et al*. (2008)
	*G.* species D (PM)	G. spD	Mal	KF242361 KF242374	this study
22	*G.* species 4 (JH)	G. sp4	Mal	FJ617050 FJ617090	Herler *et al*. (2009)
23	*G. “unicolor”*	G. uni	GBR	EF540584 EF527254	Harold *et al*. (2008)
24	*P. echinocephalus*	P. ech	RS	FJ617051 FJ617091	Herler *et al*. (2009)
25	*P. xanthosoma*	P. xan	RS	FJ617053 FJ617093	Herler *et al*. (2009)

* recently described as *G. aoyagii* by Shibukawa *et al*. (2013)

**Table 2:** Body proportions of holotype and four paratypes of *Gobiodon bilineatus* sp. nov. from the northern Red Sea. Values are proportions of standard length (SL) and head length (last five measurements), respectively, means and the first standard deviation (SD). d = damaged.

Status Coll.No.	Holotype NMW 95077	Paratype NMW 95563	Paratype NMW 95564	Paratype MNHN 2012-0262	Paratype BMNH 2006.10.6.1	MEAN (±SD)
SL (mm)	35.7	32.9	22.7	34.9	29.4	
Snout to first dorsal-fin origin	36.3	36.6	34.7	34.8	38.0	36.1 (1.4)
First dorsal-fin origin to second dorsal-fin origin	22.8	26.8	23.7	22.5	23.4	23.8 (1.7)
Second dorsal-fin origin to anal-fin origin	34.6	33.2	33.7	32.7	36.0	34.0 (1.3)
Pelvic-fin origin to anal-fin origin	24.6	22.0	25.0	23.3	20.9	23.1 (1.7)
Snout to pelvic-fin origin	36.9	35.4	38.6	40.3	42.1	38.6 (2.7)
First dorsal-fin origin to pelvic-fin origin	39.8	36.1	36.2	37.0	40.0	37.8 (1.9)
First dorsal-fin origin to anal-fin origin	43.8	45.1	43.4	44.3	45.4	44.4 (0.9)
Pelvic-fin origin to second dorsal-fin origin	43.6	40.6	40.7	40.3	43.5	41.7 (1.7)
Head length	27.9	28.2	30.1	27.6	32.0	29.2 (1.9)
Head depth	32.2	32.2	34.2	30.3	34.7	32.7 (1.8)
Body depth	39.0	36.2	36.9	36.8	41.0	38.0 (2.0)
Pelvic-fin length	14.2	14.4	17.0	13.7	14.5	14.7 (1.3)
Anal-fin length	24.5	23.7	23.7	20.0	23.5	23.1 (1.8)
Second dorsal-fin length	33.9	30.6	33.9	30.8	31.5	32.2 (1.6)
Caudal-fin length	23.1	22.2	23.2	21.7	d	22.6 (0.7)
Pectoral-fin length	23.3	26.5	27.0	22.8	25.6	25.0 (1.9)
Caudal-peduncle length	23.0	23.7	22.3	24.3	23.6	23.4 (0.8)
Caudal-peduncle depth	17.9	16.0	17.9	16.3	16.5	16.9 (0.9)
Interorbital width	20.3	22.8	15.9	15.8	18.7	18.7 (3.0)
Horizontal eye diameter	22.6	22.3	25.9	23.3	22.7	23.4 (1.5)
Snout length	30.2	27.6	28.2	29.1	27.5	28.5 (1.1)
Upper-jaw length	34.3	30.8	33.8	33.2	34.5	33.3 (1.5)
First dorsal spine to first pectoral ray	70.4	60.2	53.4	62.4	57.7	60.8 (6.3)

**Table 3:** Fin counts of holotype and four paratypes of *Gobiodon bilineatus* sp. nov. from the northern Red Sea. d = damaged.

Status Coll. No.	Holotype NMW 95077	Paratype NMW 95563	Paratype NMW 95564	Paratype MNHN 2012-0262	Paratype BMNH 2006-10.6.1
D1	VI	VI	VI	VI	VI
D2	11	10	11	10	10
A	9	9	9	8	9
C (segmented)	17	17	15	15	d
C (branched)	17	17	17	17	d
P	20	19	19	20	19
V	I/5 + I/5	I/5 + I/5	I/5 + I/5	I/5 + I/5	I/5 + I/5

**Table 4:** Body proportions of holotype and three adult paratypes of *Gobiodon irregularis* sp. nov. from the northern Red Sea. The juvenile paratype NMW 95566 (SL = 16.1 mm) is excluded here. Values are proportions of standard length (SL) and head length (last five measurements), respectively, means and the first standard deviation (SD).

Status Coll.No.	Holotype NMW 95078	Paratype NMW 95565	Paratype MNHN 2006-1699	Paratype BMNH 2006.10.6.2	MEAN (±SD)
SL (mm)	30.1	23.4	32.3	29.6	
Snout to first dorsal-fin origin	37.9	35.4	36.9	38.1	37.0 (1.3)
First dorsal-fin origin to second dorsal-fin origin	24.7	23.4	24.3	24.3	24.1 (0.6)
Second dorsal-fin origin to anal-fin origin	33.8	31.7	31.5	35.1	33.0 (1.7)
Pelvic-fin origin to anal-fin origin	25.3	26.4	26.7	25.8	26.1 (0.6)
Snout to pelvic-fin origin	37.6	38.0	36.5	37.6	37.4 (0.6)
First dorsal-fin origin to pelvic-fin origin	40.0	35.1	37.5	39.2	37.9 (2.2)
First dorsal-fin origin to anal-fin origin	44.3	43.0	43.6	45.8	44.2 (1.2)
Pelvic-fin origin to second dorsal-fin origin	43.0	39.2	40.5	42.9	41.4 (1.9)
Head length	31.1	28.7	30.2	30.9	30.2 (1.1)
Head depth	34.2	30.9	31.2	34.5	32.7 (1.9)
Body depth	39.0	35.1	35.7	38.4	37.0 (1.9)
Pelvic-fin length	18.1	17.3	16.5	18.3	17.6 (0.8)
Anal-fin length	22.8	22.5	21.9	24.1	22.8 (0.9)
Second dorsal-fin length	32.3	31.5	29.4	32.7	31.5 (1.5)
Caudal-fin length	24.6	24.2	22.6	24.3	23.9 (0.9)
Pectoral-fin length	26.5	24.7	24.9	25.8	25.5 (0.9)
Caudal-peduncle length	21.8	21.5	22.1	22.5	22.0 (0.4)
Caudal-peduncle depth	15.4	14.5	14.6	15.8	15.1 (0.6)
Interorbital width	21.3	15.6	18.1	17.5	18.1 (2.4)
Horizontal eye diameter	23.3	25.6	24.3	23.5	24.2 (1.0)
Snout length	31.0	29.6	32.9	32.4	31.5 (1.5)
Upper-jaw length	36.6	38.2	34.4	34.7	36.0 (1.8)
First dorsal spine to first pectoral ray	58.6	54.0	53.2	56.1	55.5 (2.4)

**Table 5:** Fin counts of holotype and four paratypes of *Gobiodon irregularis* sp. nov. from the northern Red Sea. d = damaged.

Status Coll. No.	Holotype NMW 95078	Paratype NMW 95565	Paratype NMW 95566	Paratype MNHN 2006-1699	Paratype BMNH 2006-10.6.2
D1	VI	VI	VI	VI	VI
D2	11	11	11	10	11
A	9	10	9	9	9
C (segmented)	16	17	d	15	16
C (branched)	17	17	d	17	17
P	20	20	20	20	20
V	I/5 + I/5	I/5 + I/5	I/5 + I/5	I/5 + I/5	I/5 + I/5

**Table 6:** Body proportions of holotype and five paratypes of *Gobiodon ater* sp. nov. from the northern Red Sea. Values are proportions of standard length (SL) and head length (last five measurements), respectively, means and the first standard deviation (SD). d = damaged.

Status Coll.No.	Holotype NMW 94612	Paratype NMW 94613	Paratype BMNH 2012.3.20.1	Paratype BMNH 2012.3.20.2	Paratype MNHN 2012-0110	Paratype MNHN 2012-0111	MEAN (±SD)
SL (mm)	26.5	26.4	23.1	24.6	25.4	15.9	
Snout to first dorsal-fin origin	36.7	38.0	37.7	36.9	37.5	41.8	38.4 (2.0)
First dorsal-fin origin to second dorsal-fin origin	25.7	26.6	24.6	25.1	26.8	24.5	25.5 (1.1)
Second dorsal-fin origin to anal-fin origin	31.5	30.7	32.3	30.8	29.5	29.2	30.5 (1.2)
Pelvic-fin origin to anal-fin origin	31.1	31.7	30.6	30.4	34.3	25.1	30.4 (3.3)
Snout to pelvic-fin origin	37.9	38.2	40.3	38.5	38.5	39.3	39.0 (0.9)
First dorsal-fin origin to pelvic-fin origin	40.0	39.9	39.2	39.2	40.6	37.0	39.2 (1.4)
First dorsal-fin origin to anal-fin origin	46.5	46.4	45.6	45.5	46.7	42.7	45.4 (1.6)
Pelvic-fin origin to second dorsal-fin origin	45.5	44.9	42.2	44.0	45.3	39.8	43.2 (2.3)
Head length	29.1	29.5	31.4	30.0	28.9	31.7	30.3 (1.2)
Head depth	33.0	32.6	33.4	32.1	33.8	29.3	32.2 (1.8)
Body depth	40.1	41.2	39.7	39.3	40.5	37.0	39.5 (1.6)
Pelvic-fin length	15.4	15.9	17.5	16.4	17.9	18.2	17.2 (1.0)
Anal-fin length	19.4	17.2	19.4	19.2	17.2	19.1	18.4 (1.1)
Second dorsal-fin length	29.3	30.5	29.0	28.6	28.9	29.4	29.3 (0.7)
Caudal-fin length	20.2	21.2	23.0	21.8	20.9	22.7	21.9 (0.9)
Pectoral-fin length	19.1	19.7	d	23.0	22.6	25.0	22.6 (2.2)
Caudal-peduncle length	22.8	21.6	22.5	21.8	20.3	21.7	21.6 (0.8)
Caudal-peduncle depth	14.2	13.8	14.3	14.7	13.9	14.5	14.2 (0.4)
Interorbital width	16.3	15.0	17.0	16.3	16.3	17.9	16.5 (1.0)
Horizontal eye diameter	19.3	20.9	21.4	19.3	21.3	23.4	21.3 (1.5)
Snout length	27.8	26.6	29.2	26.6	28.3	28.0	27.7 (1.1)
Upper-jaw length	34.9	36.6	36.4	35.1	38.0	36.5	36.5 (1.0)
First dorsal spine to first pectoral ray	51.5	53.9	52.1	54.3	62.7	48.4	54.3 (5.2)

**Table 7:** Fin counts of holotype and five paratypes of *Gobiodon ater* sp. nov. from the northern Red Sea.

Status Coll.No.	Holotype NMW 94612	Paratype NMW 94613	Paratype BMNH 2012.3.20.1	Paratype BMNH 2012.3.20.2	Paratype MNHN 2012-0110	Paratype MNHN 2012-0111
D1	VI	VI	VI	VI	VI	VI
D2	10	10	10	10	10	10
A	8	8	8	8	8	8
C (segmented)	17	17	17	17	16	17
C (branched)	17	17	17	17	16	17
P	19	19	19	19	20	19
V	I/5+I/5	I/5+I/5	I/5+I/5	I/5+I/5	I/5+I/5	I/5+I/5

**Table 8:** Body proportions of holotype and seven paratypes of *Gobiodon fuscoruber* sp. nov. from the Red Sea and western Pacific. Values are proportions of standard length (SL) and head length (last five measurements), respectively, means and the first standard deviation (SD); d = damaged.

Status Coll.No.	Holo-type NMW 95079	Para-type NMW 95080	Para-type MNHN 2006- 1700	Para-type BMNH 1951-1- 16-554	Para-type BMNH 1951-1- 16-555	Para-type OMNH 39984	Para-type OMNH 39986	Para-type OMNH 39990	MEAN (±SD)
SL (mm)	36.7	31.3	32.1	29.2	34.0	29.2	23.6	28.5	
Snout to first dorsal-fin origin	39.8	41.1	42.6	40.3	38.2	37.4	36.5	38.2	39.3 (2.0)
First dorsal-fin origin to second dorsal-fin origin	25.5	24.5	25.2	21.9	21.9	21.3	22.1	23.2	23.2 (1.6)
Second dorsal-fin origin to anal-fin origin	36.9	35.2	37.2	35.0	33.6	32.9	32.2	33.1	34.5 (1.9)
Pelvic-fin origin to anal-fin origin	25.2	25.0	25.4	29.8	29.1	28.2	28.5	26.5	27.2 (1.9)
Snout to pelvic-fin origin	39.9	42.0	40.9	38.1	39.1	38.0	38.1	41.6	39.7 (1.6)
First dorsal-fin origin to pelvic-fin origin	42.7	42.1	44.3	41.2	40.4	39.0	38.5	41.4	41.2 (1.9)
First dorsal-fin origin to anal-fin origin	48.5	47.5	47.7	47.6	46.0	42.9	44.4	45.1	46.2 (2.0)
Pelvic-fin origin to second dorsal-fin origin	45.3	43.9	46.0	44.1	43.4	43.4	42.4	43.5	44.0 (1.1)
Head length	29.6	32.4	32.0	31.1	29.1	28.1	28.3	29.3	30.0 (1.7)
Head depth	35.5	33.8	36.2	34.0	35.1	35.4	34.4	38.4	35.3 (1.5)
Body depth	42.9	40.9	43.8	40.9	40.1	39.6	38.3	41.2	41.0 (1.7)
Pelvic-fin length	16.1	16.4	18.3	16.6	17.0	16.1	16.4	15.4	16.5 (0.8)
Anal-fin length	21.1	20.3	20.5	19.3	20.1	21.2	21.1	21.9	20.7 (0.8)
Second dorsal-fin length	27.9	28.8	30.1	27.8	28.7	31.5	31.3	28.1	29.3 (1.5)
Caudal fin length	23.4	24.6	23.7	23.8	d	22.4	19.1	20.4	22.5 (2.0)
Pectoral-fin length	26.6	25.4	26.2	d	d	26.4	26.9	24.2	26.0 (1.0)
Caudal-peduncle length	21.4	22.2	21.9	22.9	23.7	21.6	22.5	21.2	22.2 (0.8)
Caudal-peduncle depth	15.7	14.8	15.4	16.0	15.2	14.1	15.6	15.4	15.3 (0.6)
Interorbital width	18.5	14.9	16.4	17.0	17.6	17.4	18.6	17.1	17.2 (1.2)
Horizontal-eye diameter	20.2	18.1	19.0	18.9	19.0	19.9	21.1	19.9	19.5 (0.9)
Snout length	29.7	31.9	33.9	29.8	30.1	27.3	26.2	26.0	29.4 (2.8)
Upper-jaw length	36.0	32.0	36.7	29.8	30.7	28.5	28.0	31.8	31.7 (3.2)
First dorsal spine to first pectoral ray	69.2	62.1	62.7	59.8	63.3	50.1	50.5	55.5	59.2 (6.6)

**Table 9:** Fin counts of holotype and seven paratypes of *Gobiodon fuscoruber* sp. nov. from the Red Sea and western Pacific.

Status Coll.No.	Holotype NMW 95079	Paratype NMW 95080	Paratype MNHN 2006-1700	Paratype BMNH 1951-1-16-554	Paratype BMNH 1951-1-16-555	Paratype OMNH 39984	Paratype OMNH 39986	Paratype OMNH 39990
D1	VI	VI	VI	VI	VI	VI	VI	VI
D2	10	10	10	10	10	10	10	10
A	8	8	8	8	8	8	8	8
C (br.)	17	17	17	17	17	17	17	17
C (segm.)	17	17	17	17	17	17	17	17
P	20	20	20	19	20	19	19	19
V	I/5+I/5	I/5+I/5	I/5+I/5	I/5+I/5	I/5+I/5	I/5+I/5	I/5+I/5	I/5+I/5

**Table 10:** Selection of genetic (*p*-)distances from pairwise comparisons of specimens of *G. bilineatus* sp. nov., *G. irregularis* sp. nov., *G. ater* sp. nov. and *G. fuscoruber* sp. nov. from different regions (GBR: Great Barrier Reef, Jap: Japan, Mal: Maldives, RS: Red Sea, Tai: Taiwan). For comparison of intra- and interspecific genetic distances across the genus, other species from different regions as well as “*G. unicolor*” (sensu Munday *et al*. (1999) and Harold *et al*. (2008)) from the GBR are included. Italic numbers indicate intraspecific distances. Bold values indicate intraspecific distances of more than 2%. For species abbreviations and Genbank accession numbers see Table 1.

	*G. cit* RS	*G. his* RS	*G. riv* RS	*G. bil* RS	*G. irr* RS	*G. ate* RS	*G. fus* RS	*G. fus* Mal	*G. fus* Jap
*G. cit* GBR	***0.031***								
*G. his* GBR		*0.005*							
*G. riv* GBR			***0.023***						
*G. bil* Mal				*0.011*					
*G. bil* Tai				*0.014*					
*G. qui* Mal				0.033					
*G. ret* RS					0.026				
*G. ate* Mal						*0.016*			
*G. ate* Tai						*0.013*			
*G. fus* RS						0.023			
*G. fus* Mal						0.026	*0.019*		
*G. fus* Jap						0.031	*0.019*	*0.016*	
“*G. uni*” GBR						0.032	*0.020*	*0.017*	*0.001*
